# Anti-Inflammatory Potential of 1-Aryl-6,7-Dimethoxy-1,2,3,4-Tetrahydroisoquinolines: Structure–Activity Relationship and COX-2 Binding

**DOI:** 10.3390/molecules31111956

**Published:** 2026-06-04

**Authors:** Azizbek A. Azamatov, Firuza M. Tursunkhodzhaeva, Sherzod N. Zhurakulov, Zufar D. Boboev, Kuvonchbek F. Kuchimov, Urkhiya K. Aytmuratova, Ilhomjon S. Ortikov, Robiya Sh. Abdurazakova, Valentina I. Vinogradova, Izzatullo Z. Abdullaev, Ulugbek G. Gayibov

**Affiliations:** 1 Institute of the Chemistry of Plant Substances, Academy of Sciences of the Republic of Uzbekistan, M. Ulugbek Str 77, Tashkent 100170, Uzbekistan; azizbek.azamatov@bk.ru (A.A.A.); j.sherzod.78@mail.ru (S.N.Z.); quvonchkoch98@gmail.com (K.F.K.); urxiyaaytmuratova@mail.ru (U.K.A.); abdurazakova.robiyaaa@gmail.com (R.S.A.); cnc@icps.org.uz (V.I.V.); 2 Faculty of Pharmacy and Chemistry, Alfraganus University, Yuqori Qoraqamish Str. 2a, Tashkent 100190, Uzbekistan; zufarbek1313@gmail.com (Z.D.B.); ortikovilxomjon@gmail.com (I.S.O.); 3 Institute of Bioorganic Chemistry, Academy of Sciences of the Republic of Uzbekistan, M. Ulugbek Str 87, Tashkent 100143, Uzbekistan; izzatillo.abdullayev.00@mail.ru (I.Z.A.); gayibov.ulugbek@gmail.com (U.G.G.)

**Keywords:** 1-aryl-1,2,3,4-tetrahydroisoquinoline, anti-inflammatory activity, structure-activity, COX-2

## Abstract

Non-steroidal anti-inflammatory drugs (NSAIDs) are used globally for their pain-relieving and fever-reducing properties. However, excessive intake of NSAIDs can have harmful effects on multiple body systems, including the cardiovascular, gastrointestinal, hepatic, renal, and nervous systems. The anti-inflammatory activity of 34 derivatives of 1-aryl-6,7-dimethoxy-1,2,3,4-tetrahydroisoquinoline was investigated in vivo. A relationship between the activity of the compounds and the nature of their substituents, as well as their positional and mutual arrangement in the C ring (1-Ar-), was established. In silico modeling of these 1-aryl-6,7-dimethoxy-1,2,3,4-tetrahydroisoquinoline derivatives’ interaction with the COX-2 (PDB ID: 1PXX) active site revealed that the nitro-derivatives exhibited the highest stability owing to their superior capacity for electrostatic and hydrogen bond formation compared to brominated compounds. These data on the effects of the substituents –NH_2_, –OH, and –OCH_3_ in ring C (1-Ar-) of 1-aryl-6,7-dimethoxy-1,2,3,4-tetrahydroisoquinolines on anti-inflammatory activity promote the search for new, highly effective derivatives within this series.

## 1. Introduction

**Figure 1:**
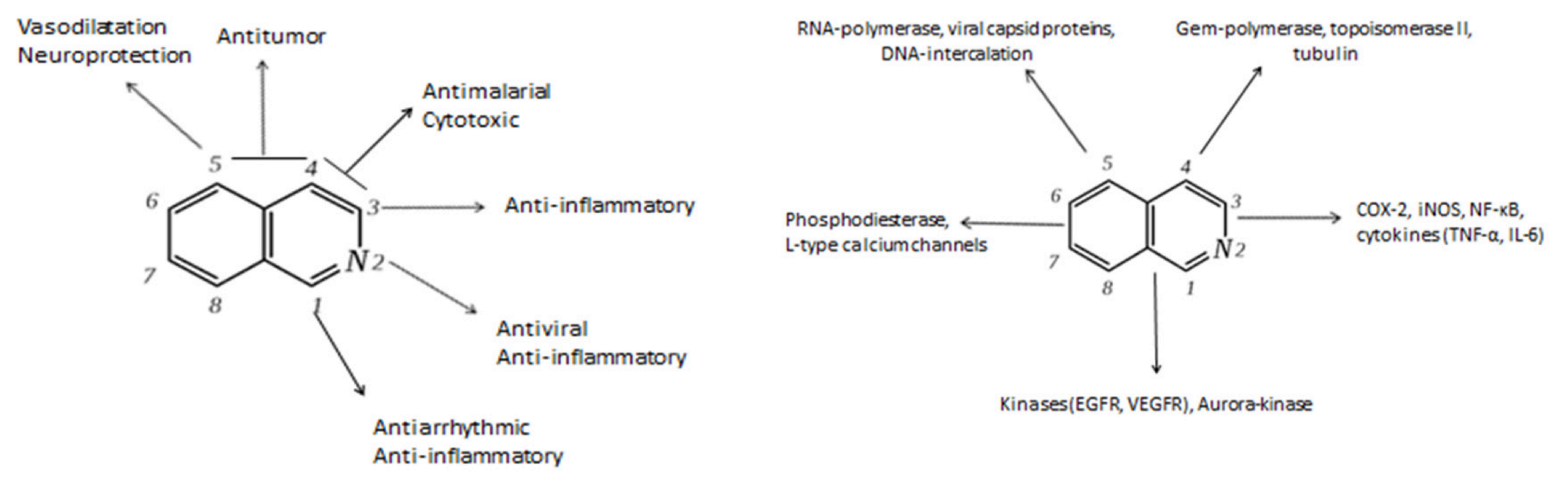
Complex profile of isoquinoline scaffold: positions, targets, and biological activity [4,5,6,7,8].

Isoquinoline derivatives exhibit a wide spectrum of biological activity. Among these derivatives are compounds with antitumor, antidiabetic, antiviral, anti-inflammatory, immunosuppressive, antiparasitic, neuroprotective, hepatoprotective, and cardioprotective effects (Figure 1) [1]. Several 1,2,3,4-tetrahydroisoquinoline derivatives have demonstrated analgesic, antipyretic, anticholinesterase, antioxidant, and anticonvulsant activities [2,3].

The diisoquinoline alkaloid berberine exhibits pronounced anti-inflammatory activity, the mechanism of which is associated with the regulation of pro- and anti-inflammatory cytokines [9]. Demethyleneberberine, which differs from berberine by the presence of a 2,3-dioxy group instead of a 2,3-methylenedioxy group, reduces inflammatory responses by inhibiting the NF-κB pathway and regulating the Th-cell balance [10]. Among aporphine alkaloids, thalicmidine possesses anti-inflammatory properties, while norisoboldine exerts anti-arthritic effects through anti-inflammatory and immunoregulatory mechanisms [11]. The bisbenzylisoquinoline alkaloid curine has been shown to inhibit the inflammatory phase II in the formalin test, reduce the number of pain responses in the acetic acid writhing test, and additionally decrease the production of prostaglandin E2 [12].

Recent studies have focused on identifying moderately selective cyclooxygenase-2 (COX-2) inhibitors in order to overcome the adverse effects of highly selective COX-2 and non-selective COX-2 inhibitors. In [13,14], hybrids of 1,2,4-triazole and tetrahydroisoquinoline were synthesized and demonstrated anti-COX-2 activity comparable to that of celecoxib (IC_50_ from 0.58 to 1.27 μM). These compounds significantly reduced levels of key inflammatory mediators such as prostaglandin E2 (PGE-2), tumor necrosis factor (TNF-α), and interleukin-6 (IL-6).

The analgesic and anti-inflammatory activities of 1-(4′-dimethylaminophenyl)-6,7-dimethoxy-1,2,3,4-tetrahydroisoquinoline hydrochloride were studied under conditions of thermal (hot plate test) and chemical (acetic writhing test) irritation and in a model of acute inflammatory arthritis, respectively. The compound showed a pronounced anti-inflammatory effect that was 3.3 times greater than that of diclofenac sodium at a dose of 0.5 mg/kg [3].

New hybrid structures exhibiting dual agonist/antagonist properties at the κ- and μ-opioid receptors have been developed based on TGIQ-valine [15,16,17].

A current research trend is the development of hybrid compounds (for example, with Quercetin) that combine analgesic activity with antioxidant and neuroprotective effects. Such compounds are considered promising agents for the management of pain associated with neurodegenerative diseases such as Alzheimer’s disease [18].

Newly obtained hybrids of ibuprofen with 1,2,3,4-tetrahydroisoquinoline and piperidine were screened for their antioxidant and antitryptic activity and ability to inhibit albumin denaturation in vitro. Their lipophilicity was established using both reversed-phase thin layer chromatography and in silico calculations [19].

Thus, the search for anti-inflammatory compounds among isoquinoline derivatives is a promising research direction.

In our previous studies [15,16], we synthesized, characterized, evaluated the purity as well as investigated the toxicity of 30 derivatives of 1-aryl-1,2,3,4-tetrahydroisoquinolines. We also studied their local anesthetic activity and analyzed structure–toxicity and structure–activity relationships. We further established that TGIQ 1-(4′-Dimethylaminophenyl)-6,7-dimethoxy-1,2,3,4-tetrahydroisoquinoline hydrochloride alone has strong analgesic and anti-inflammatory effects [3]. In continuation of this research, we conducted this study to investigate the in vivo anti-inflammatory effects and to evaluate COX-2-binding in silico and the structure–activity relationships of thirty-three derivatives of 1-aryl-1,2,3,4-tetrahydroisoquinoline. These studies are essential for predicting the anti-inflammatory effects and potential side effects of the substances investigated, which may serve as a basis for the development of new, highly effective non-steroidal anti-inflammatory drugs. In silico studies were also conducted by applying molecular docking simulations to investigate the binding of the tested substances at the binding site of cyclooxygenase-2 (COX-2) enzyme (PDB ID: 1PXX). The structure–activity relationships of the compounds were studied to determine the essential structural features necessary for their anti-inflammatory activity. A predictive ADMET study was conducted using Discovery Studio 4.0 software to determine the pharmacokinetic profiles and evaluate the drug-likeness of the tested compounds. The results, based on their chemical structures, showed a strong correlation with various parameters, such as aqueous solubility, absorption level, blood–brain barrier permeability, hepatotoxicity probability, atom-based log P98 (A LogP98), plasma protein binding (PPB) level, cytochrome P450 2D6 (CYP2D6), and 2D polar surface area (ADMET 2D PSA).

## 2. Results

### 2.1. Synthesis of the Compounds

**Scheme 1:**
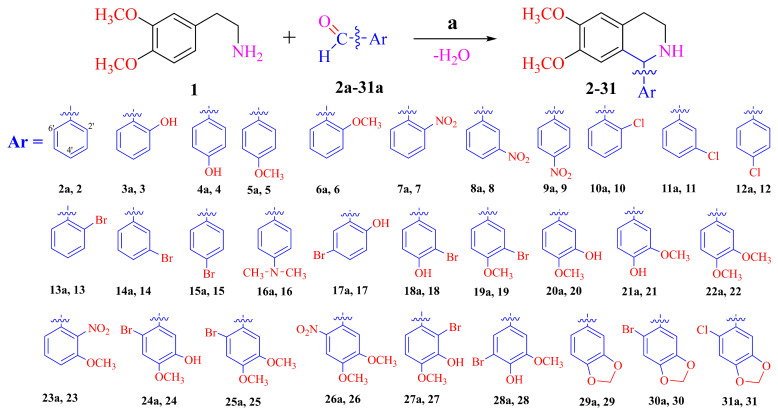
Synthesis of 1-aryl-6,7-dimethoxy-1,2,3,4-tetrahydroisoquinoline derivatives **2**–**31**, (a: CF_3_COOH, reflux 4–6 h).

**Scheme 2:**
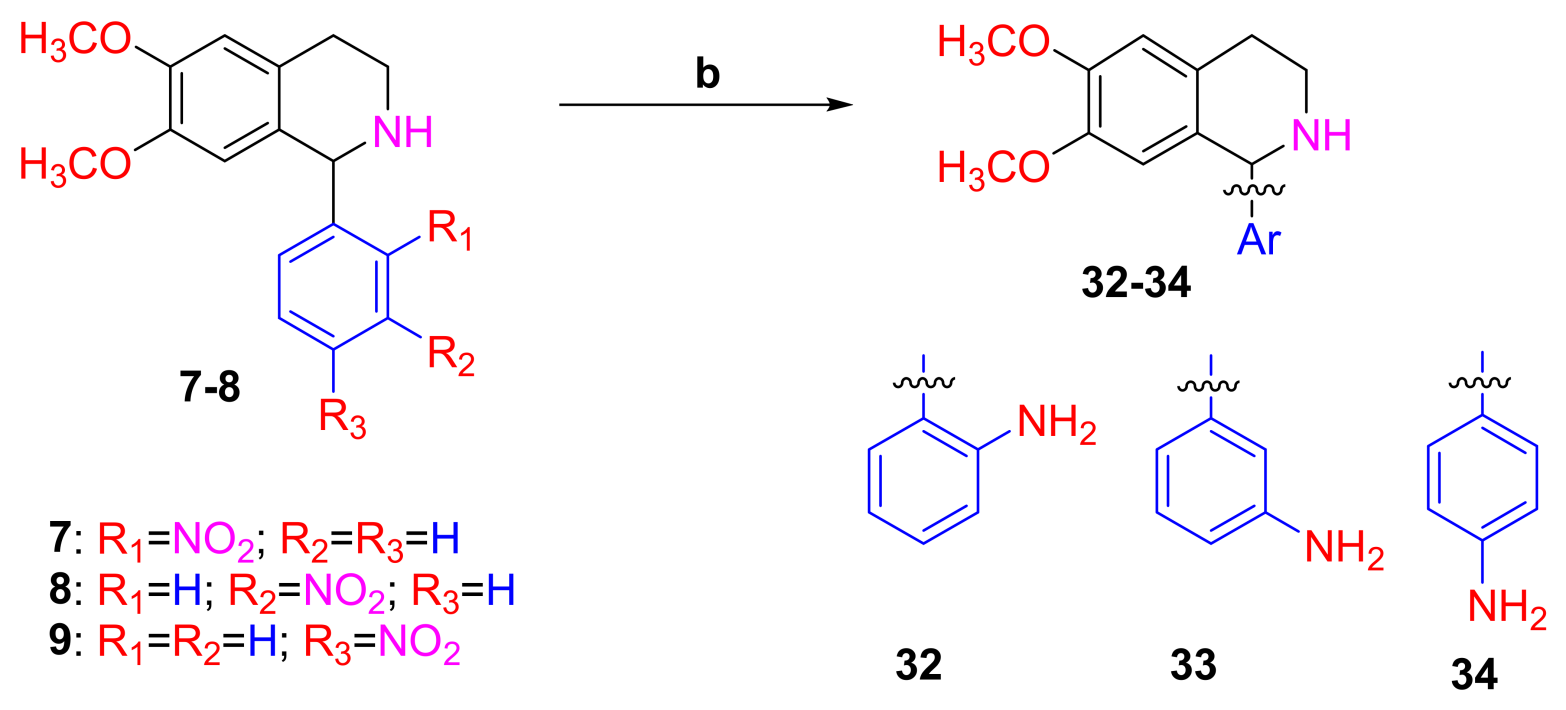
Reduction of 1-(2′-nitrophenyl)—(**7**), 1-(3′-nitrophenyl)—(**8**), 1-(3′-nitrophenyl)-6,7-dimethoxy-1,2,3,4-tetrahydroisoquinolines (**9**), resulting in the formation of derivatives **32**–**34**, (b: SnCl_2_×2H_2_O, HCl, C_2_H_5_OH, reflux, 4 h).

Series of derivatives of 1-aryl-1,2,3,4-tetrahydroisoquinoline (Scheme 1 and Scheme 2) were obtained as described in [15,16].

### 2.2. Biological Evaluation

#### 2.2.1. Anti-Inflammatory Effects in a Formalin Rat Model

An animal study of the effects of 1-aryl-1,2,3,4-tetrahydroisoquinolines (Table 1) showed that at a dose of 1 mg/kg, substances **33**, **23**, **15**, **16** and **19** were the most effective and demonstrated a greater effect (AIE) on the acute stage of inflammation compared to the reference drugs ketoprofen and sodium diclofenac. The anti-inflammatory effects (AIE) and cytokine levels were investigated in vivo at doses of 1–10 mg/kg, and the most effective dose (1 mg/kg) was selected based on the results of the experiments (Table 1, Table 2 and Table S1).

The effects of the most active compounds, **33**, **23**, **15**, **16** and **19**, on cytokine levels showed that, under the influence of these compounds, the high levels of interleukins IL-4, IL-6, and TNF in rats with formalin-induced inflammation reached the levels found in intact animals (Table 2).

#### 2.2.2. Anti-Inflammatory Effects in Carrageenan Rat Model

Prostaglandin E2 is a key pro-inflammatory mediator produced from arachidonic acid by COX-2, particularly during inflammation, cancer, and pain. COX-2 inhibitors (NSAIDs) reduce PGE_2_ levels to alleviate inflammation and pain but can cause side effects by blocking protective prostanoids [20]. To evaluate the inhibiting effects of the examined compounds on COX-2, we investigated their effects on prostaglandin E_2_ (PGE_2_) levels in the serum of experimental animals. Compounds **2**, **6**, **7**, **8**, **10**, **11**, **13**, **14**, **15**, **16**, **18**, **19**, **20**, **21**, **23**, **24**, **26**, **29**, **31**, **32**, **33**, and **34**, which reduced the level of PGE_2_ to ≤500 pg/mL, were considered highly effective substances (Table 3 and Table S2). In particular, compounds **33**, **32**, **34**, **10**, **7**, and **23** showed the strongest effect, suppressing the synthesis of PGE_2_ to a level close to or even higher than that achieved with the reference drug sodium diclofenac, indicating that these substances have a significant effect on the formation of COX-related inflammatory prostaglandins.

The moderately effective compounds showed anti-inflammatory effects but did not reach maximum activity. The PGE_2_ levels in serum treated with compounds **3**, **4**, **5**, **25**, and **28** were maintained in the range of 501–600 pg/mL. These results indicate that although these compounds have anti-inflammatory activity, they are less effective at inhibiting prostaglandin E_2_ biosynthesis than those in the highly effective group. Therefore, these compounds may be pharmacologically promising, but they need to be optimized or structurally modified.

The low-efficacy compounds did not sufficiently reduce PGE_2_ levels. The PGE_2_ levels in serum treated with compounds **9**, **12**, **17, 22**, **27**, and **30** remained in the range of 603.1–698.8 pg/mL. This indicates that their effects on prostaglandin E_2_ synthesis in carrageenan-induced inflammation are relatively weak. These results confirm that not all tetrahydroisoquinoline derivatives have the same pharmacological activity and that their efficacy may depend on the chemical structure and location of their substituents.

The changes in the level of TXB_2_—the specific product of COX-1—were investigated in the carrageenan-induced inflammation mode (Table 4 and Table S3). The results show that the level of TXB_2_ in the control group was significantly increased by 174% compared to the intact group, indicating that thromboxane synthesis was dramatically activated during the inflammatory process. These results demonstrate the activation of the COX-1 pathway and the enhancement of platelet aggregation in the carrageenan-induced inflammation model.

Sodium diclofenac, used as a reference drug, showed anti-inflammatory effect, reducing TXB_2_ levels by 45.0–52.1%. This result served as an evaluation criterion for the tested substances.

The tested substances differed significantly in their pharmacological efficacy. Of the 33 compounds studied, 12 showed high, 13 moderate, and 8 low efficacy.

The highly effective compounds—**6**, **7**, **8**, **10**, **15**, **20**, **23**, **26**, **31**, **32**, **33** and **34**—showed results close to or even superior to the reference drug, reducing TXB_2_ levels by 28–35%. In particular, compounds **33** (28%), **10** (30.3%) and **7** (30.9%) showed the strongest effects, indicating their ability to effectively suppress thromboxane biosynthesis.

The moderately effective compounds—**2**, **3**, **4**, **11**, **13**, **14**, **16**, **18**, **21**, **24**, **25**, **28** and **29**—reduced TXB_2_ levels by 40–60% and showed anti-inflammatory effects, but did not achieve maximum efficacy.

The low-efficacy agents—**5**, **9**, **12**, **17**, **19**, **22**, **27**, and **30**—failed to sufficiently reduce TXB_2_ levels (61–98%), indicating that their effect on thromboxane synthesis is weak.

Overall, the obtained results regarding the effects of the examined substances on COX-1 and COX-2 product levels suggest that only compounds **5** and **19** are able to inhibit COX-2 selectively. Other substances exhibit either high or moderate inhibiting activity against COX-1 and COX-2 products and may be considered as non-selective COX inhibitors. Substances **9**, **12**, **17**, **22**, **27**, and **30** show low inhibition of COX-1 and COX-2.

The results indicate that the pharmacological activity of tetrahydroisoquinoline derivatives is directly related to their chemical structure. In particular, the highly active compounds effectively inhibit thromboxane synthesis via the COX pathway, demonstrating strong anti-inflammatory potential along with platelet aggregation inhibition.

### 2.3. Molecular Docking

**Figure 2:**
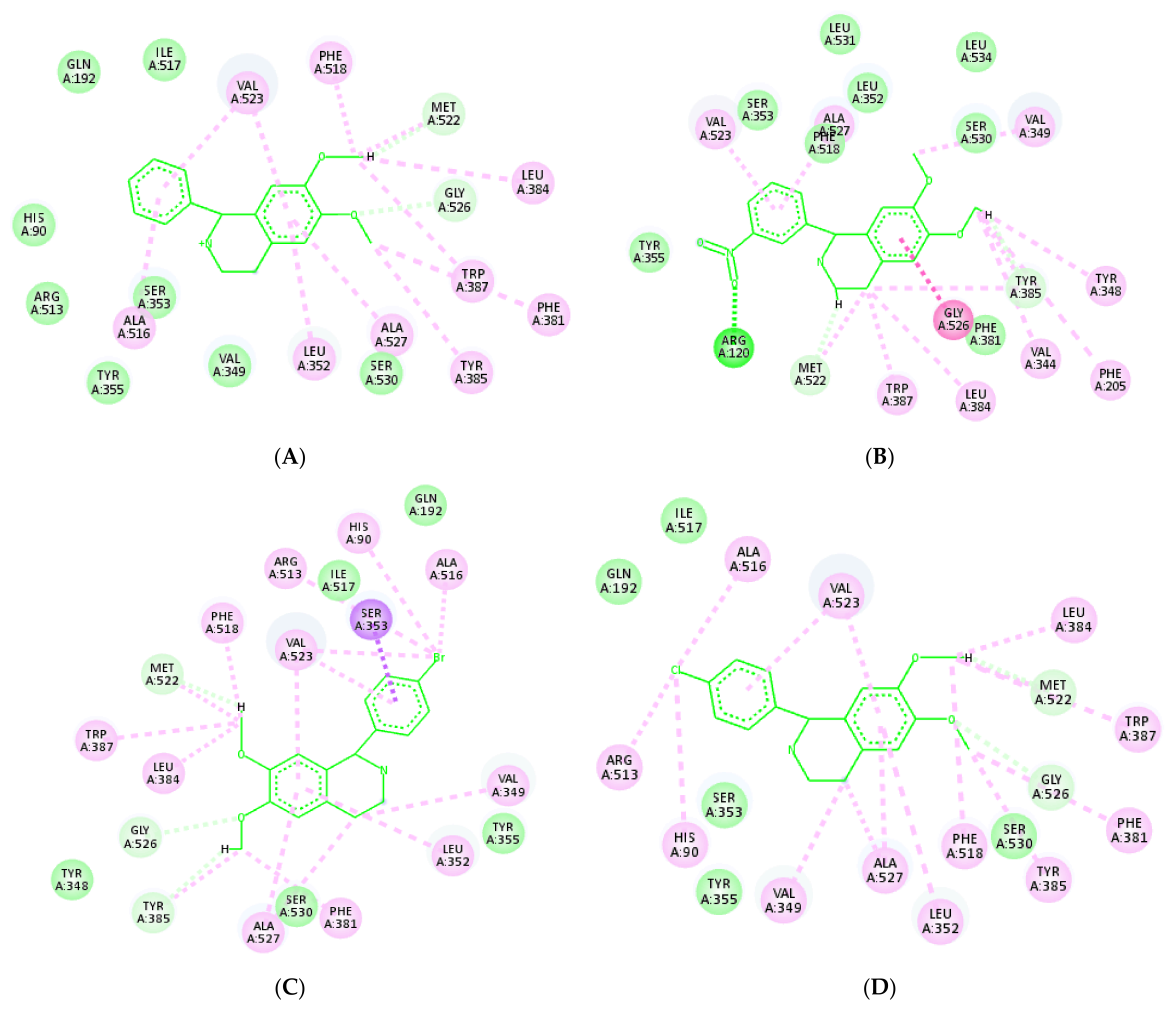
Binding interactions with amino acid residues at the active site of COX-2 (PDB ID: 1PXX). (**A**) Compound **8** (E = −9.66 Kcal/mol), (**B**) compound **2** (E = −7.83 Kcal/mol), (**C**) compound **15** (E = −8.26 Kcal/mol), and (**D**) compound **12** (E = −8.14 Kcal/mol). (Color code for interactions: H-bond: green; carbon–H bond: light blue; Pi-Sigma bond: purple; amide–pi stacked bond: pink; alkyl and Pi–alkyl bond: light pink).

The docking results of the 33 tested compounds targeting the cyclooxygenase-2 (COX-2) enzyme (PDB ID: 1PXX) [12] are presented in Table 5. The 2D interactions were compared to the downloaded ligand after re-docking, showing an RMSD value of 1.61 Å. All tested compounds exhibited comparable binding modes to the ligand (E = −7.90 Kcal/mol), with C-Docker interaction energy ranging from E = −7.83 to −9.66 Kcal/mol. Visualization revealed that the ligand formed one hydrogen bond donor (HBD) with SER530 and one pi-sulfur bond with MET522. Hydrophobic interactions were also observed, including a pi-pi T-shaped bond with TRP387 and pi–alkyl bonds with VAL349, VAL523, and ALA527. Most of the tested compounds showed similar interactions with the surrounding amino acid residues and a comparable binding mode (Table 5, Figure 2).

To evaluate the structural diversity of the synthesized series and their stability within the active site of the COX-2 enzyme, several representative compounds (**2**, **8**, **12**, and **15**) were analyzed in depth. Compound **2**, serving as the unsubstituted model, exhibited the lowest binding energy (−7.83 kcal/mol), whereas compound **8**, containing a strong electron-withdrawing nitro group (—NO_2_), displayed the highest activity in the series (−9.66 kcal/mol). Additionally, compounds **12** and **15** served as critical indicators for investigating the effects of the size and polarizability of para-substituted halogens. This selection enabled a comprehensive elucidation of the interaction mechanisms between an enzyme and substituents with varying electronic properties.

**Figure 3:**
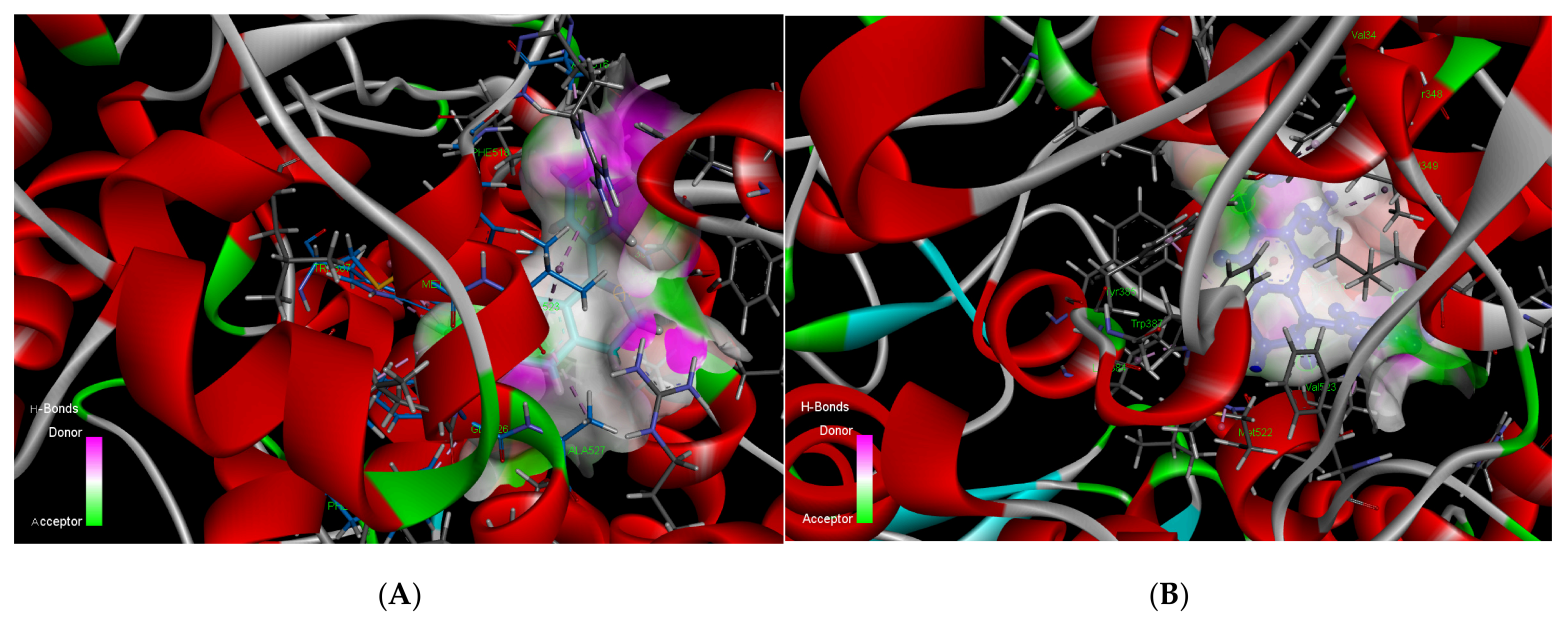
Three-dimensional molecular docking poses of the synthesized compounds within the COX-2 active site. (**A**) Compound **2** (binding affinity: −7.83 kcal/mol) serving as the baseline model with lower interaction stability. (**B**) Compound **8** (binding affinity: −9.66 kcal/mol) exhibiting the highest potency. The protein is represented in ribbon format, and the interaction pocket is highlighted with an H-bond donor (pink) and acceptor (green) surface, illustrating the superior binding fit of compound **8**.

Molecular docking simulations provided deeper insights into the binding modes of the synthesized compounds. Compound **2**, which lacks substituents on the phenyl ring, showed a binding affinity of −7.83 kcal/mol. As shown in Figure 3, it occupies the active site but lacks specific stabilizing interactions, serving as a baseline for the series. In contrast, Compound **8** exhibited the strongest affinity of −9.66 kcal/mol. The 3D visualization in Figure 2 reveals that the nitro group at the meta-position significantly enhances the binding stability. The H-bond surface analysis indicates that Compound **8** effectively utilizes both donor and acceptor regions of the COX-2 pocket, forming stronger interactions with key residues compared to the unsubstituted derivative. This correlation between docking scores and structural features supports the observed SAR trends.

### 2.4. Structure Activity Relationship

Molecular docking results were analyzed to study the essential pharmacophoric features of the studied scaffold represented as 1-aryl-6,7-dimethoxy-1,2,3,4-tetrahydroisoquinoline (1-ArTHIQ) derivatives. Compound **8** showed the best conformation with the most promising binding pose in the COX-2 binding pocket. It demonstrated superior results in terms of both the binding interactions with the key amino acid residues and the C-Docker binding interaction energy (E = −9.66 Kcal/mol), confirming a good fit with stable binding interaction exceeding that of the ligand, where two conventional hydrogen bonds, one carbon–hydrogen bond, one amide–pi stacked interaction, and several alkyl and pi–alkyl interactions occurred within the binding site (Table 4). It was determined that the position of the nitro group on the phenyl ring significantly influenced binding stability; while the meta-position (compound **8**) exhibited minimal energy, binding affinity was slightly weaker in the ortho- (compound **7**, E = −9.16 Kcal/mol) and para- (compound **9**, E = −8.69 Kcal/mol) positions. The high activity of nitro derivatives is attributed to the formation of numerous hydrogen bonds with amino acid residues. Specifically, compound **8** forms two robust hydrogen bonds with ARG120, which serves as the primary stabilizing factor for its complex. In comparison, compound **2** showed a binding energy of E = −7.83 Kcal/mol; this lower affinity is due to the absence of donor–acceptor bonds, requiring weak carbon–hydrogen and hydrophobic interactions with MET522 and GLY526 instead. The hydrogen bonds formed by compound **7** and compound **9** significantly enhance their activity compared to compound **2** [21,22].

**Figure 4:**
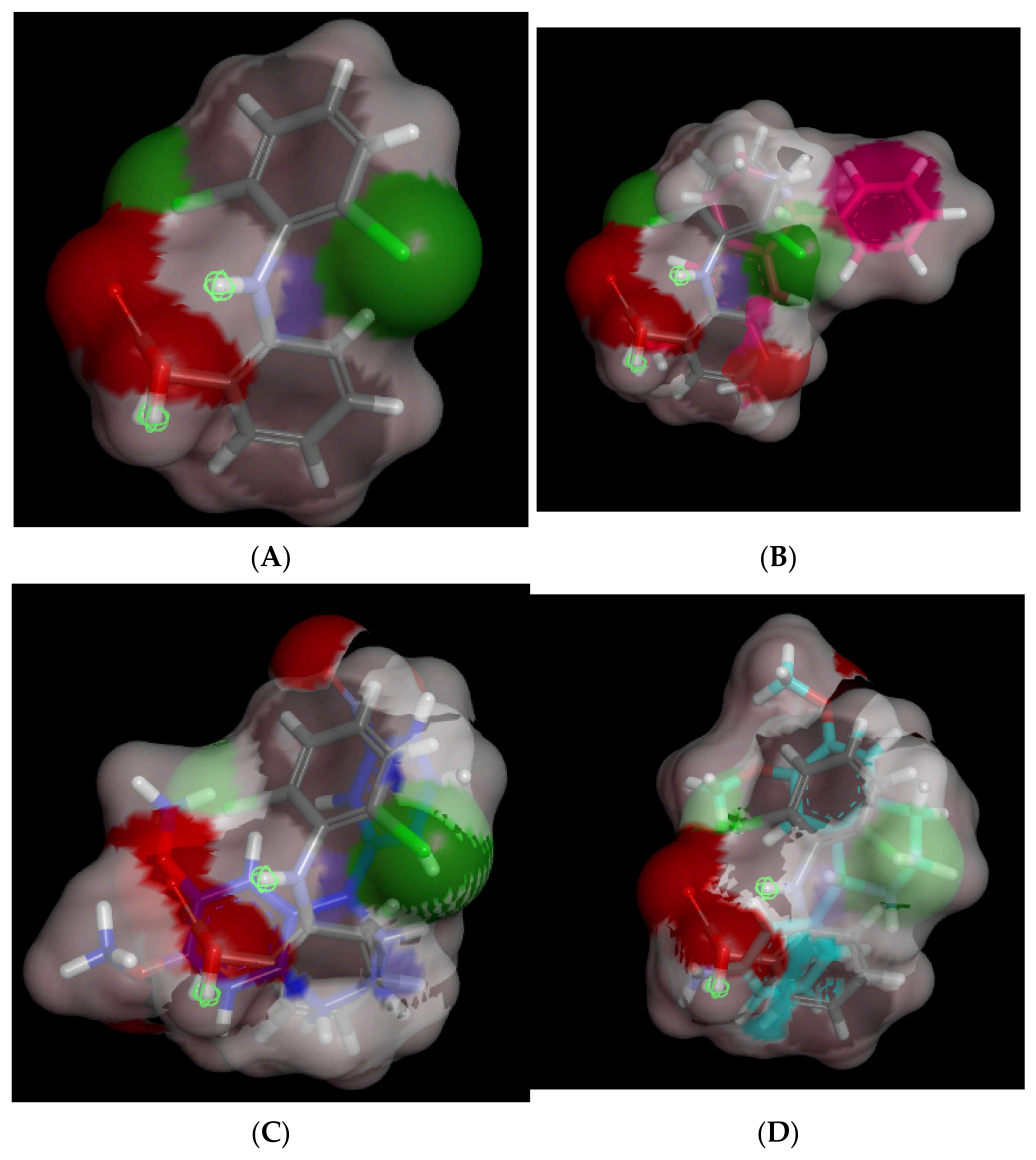
Solvent-accessible surface (SAS) representations of the reference ligand and compounds. (**A**) ligand, (**B**) overlay of ligand and compound **2** (reddish), (**C**) overlay of ligand and compound **8** (dark blue), and (**D**) overlay of ligand and compound **33** (cyan). The overlay is colored according to atom types (CPK coloring) and visualized using BIOVIA Discovery Studio.

Further modifications of the phenyl ring yielded specific results. In compound **29**, the presence of a 3,4-methylenedioxy group (E = −8.30 Kcal/mol) facilitated hydrogen bonding with HIS90 at the active site. The introduction of halogen atoms in compounds **30** and **31** further strengthened this binding. Notably, the 2-bromo substituent in compound **30** formed hydrogen and pi–alkyl bonds with TYR355, lowering the energy to E = −8.83 Kcal/mol. This value outperformed the E = −8.47 Kcal/mol recorded for the 2-chloro derivative in compound **31**. Analysis of the bromine position in compounds **13**, **14**, and **15** revealed that the meta-bromo derivative, represented by compound **14**, provided the best results with an energy of E = −8.57 Kcal/mol. This indicates that, similar to nitro derivatives, meta-substituted compounds align most effectively with the active site geometry. The superiority of nitro-containing compounds over brominated derivatives is linked to their higher capacity for electrostatic and hydrogen bond formation [23]. An overlay between the ligand and compounds **2**, **8**, and **33** at the active site is presented in Figure 4.

Docking analysis of the 1-aryl-6,7-dimethoxy-1,2,3,4-tetrahydroisoquinoline derivatives studied revealed pharmacophoric features essential for binding within the COX-2 active site. Compound **8** demonstrated the best results, forming a more stable complex compared to the ligand due to the formation of two robust hydrogen bonds with the ARG120 residue. These results indicate that the position of the nitro group on the phenyl ring significantly influences activity. Specifically, the meta-position provided the highest affinity, while the binding energy decreased slightly with the ortho and para positions. The lower activity of compound **2** was attributed to the absence of donor–acceptor bonds. Furthermore, modifications of the phenyl ring showed that the 3,4-methylenedioxy group facilitated interactions with HIS90, and the 2-bromo substituent formed additional bonds with TYR355. Overall, nitro-derivatives exhibited the highest stability owing to their superior capacity for electrostatic and hydrogen bond formation compared to brominated compounds.

### 2.5. Molecular Dynamics Simulation

To further validate the stability of the best-performing compounds obtained from docking analysis, molecular dynamics (MD) simulations were carried out for compounds **2**, **8**, **12**, and **15**. These molecules were selected from the series based on their relatively favorable docking scores and interaction profiles within the COX-2 active site. All simulations were performed for 100 ns to assess the dynamic behavior of the ligand–protein complexes over time [24].

**Figure 5:**
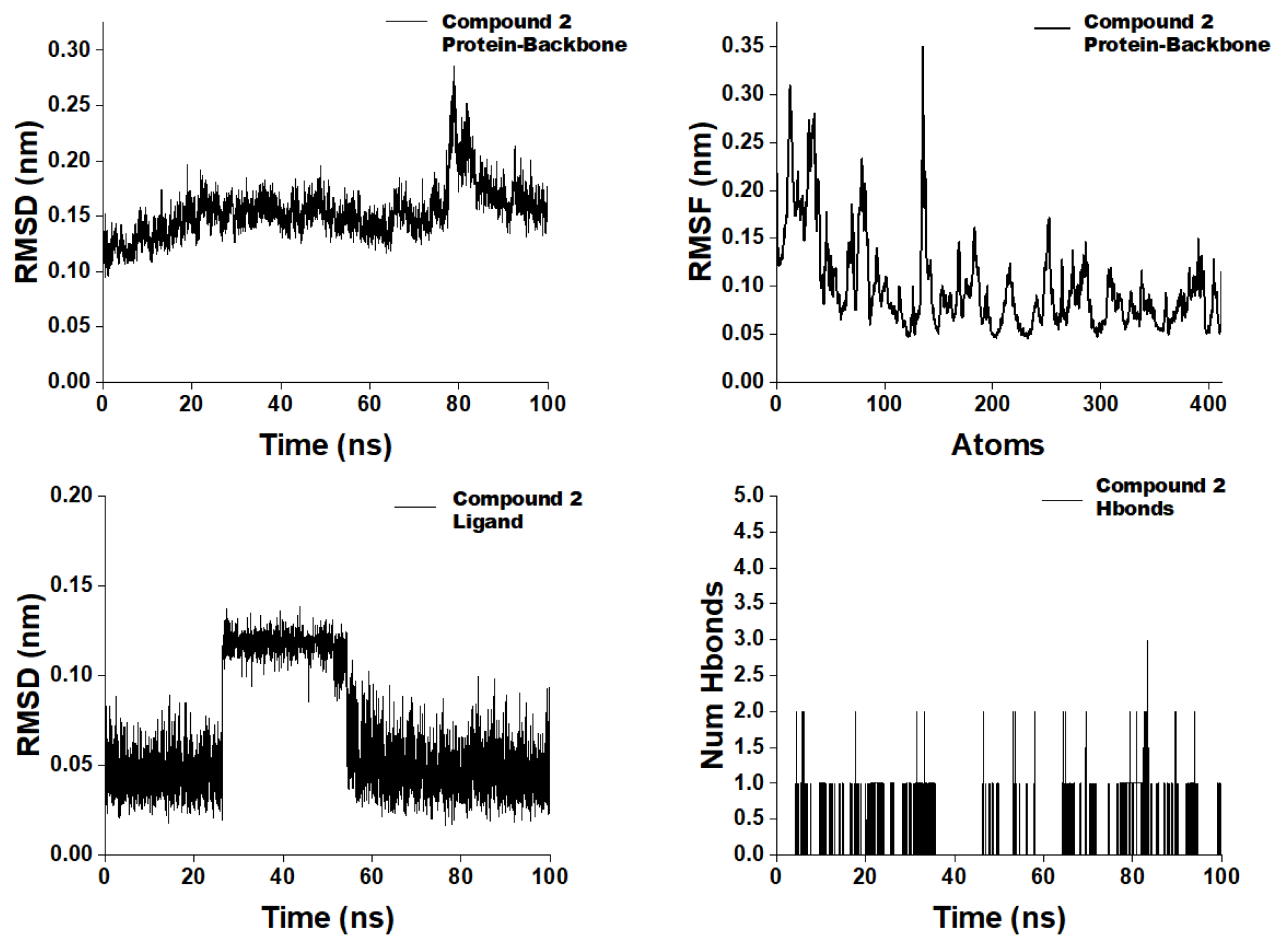
Molecular dynamics analysis of COX-2 in complex with compound **2**.

**Figure 6:**
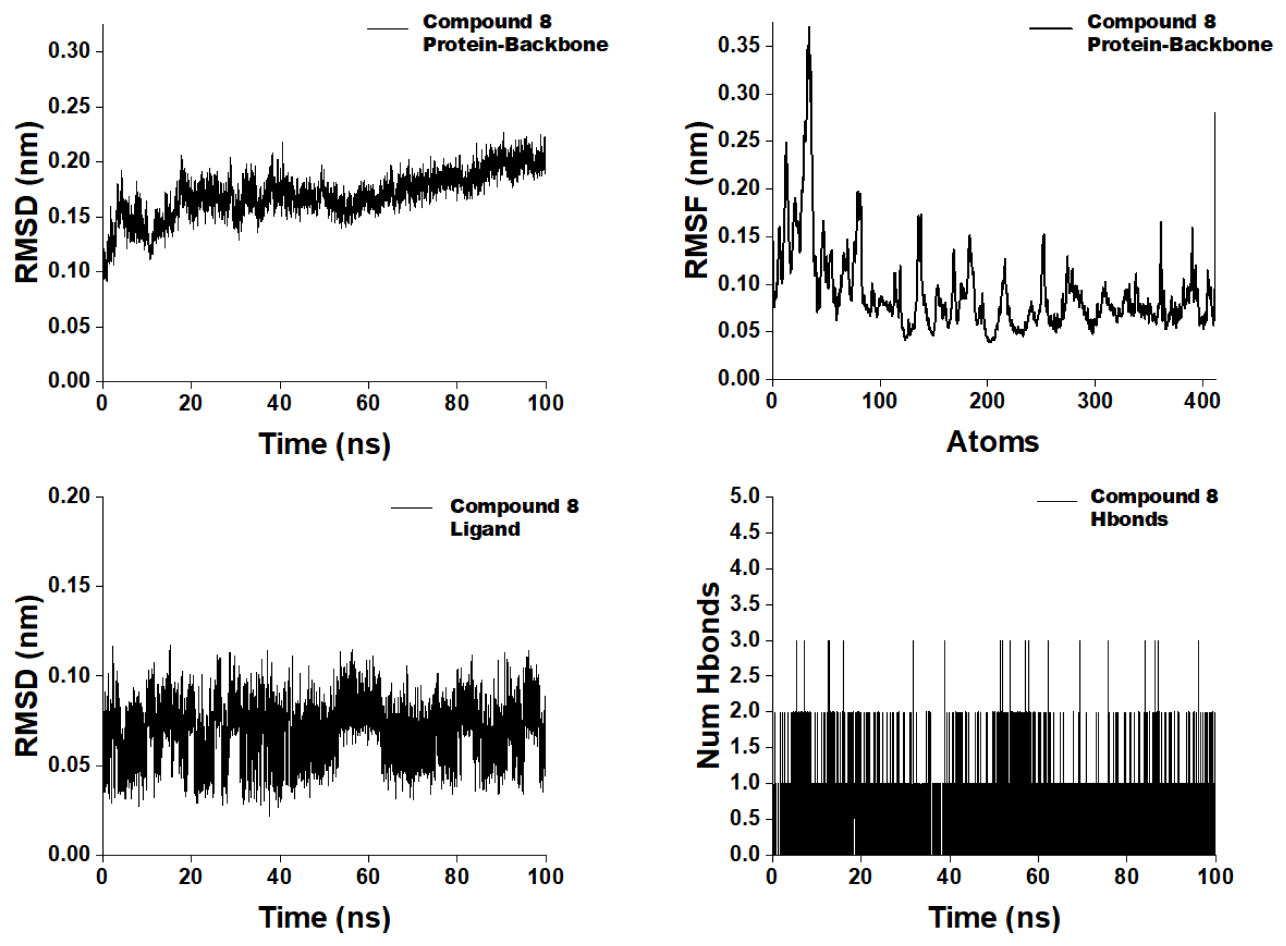
Molecular dynamics analysis of COX-2 in complex with compound **8**.

**Figure 7:**
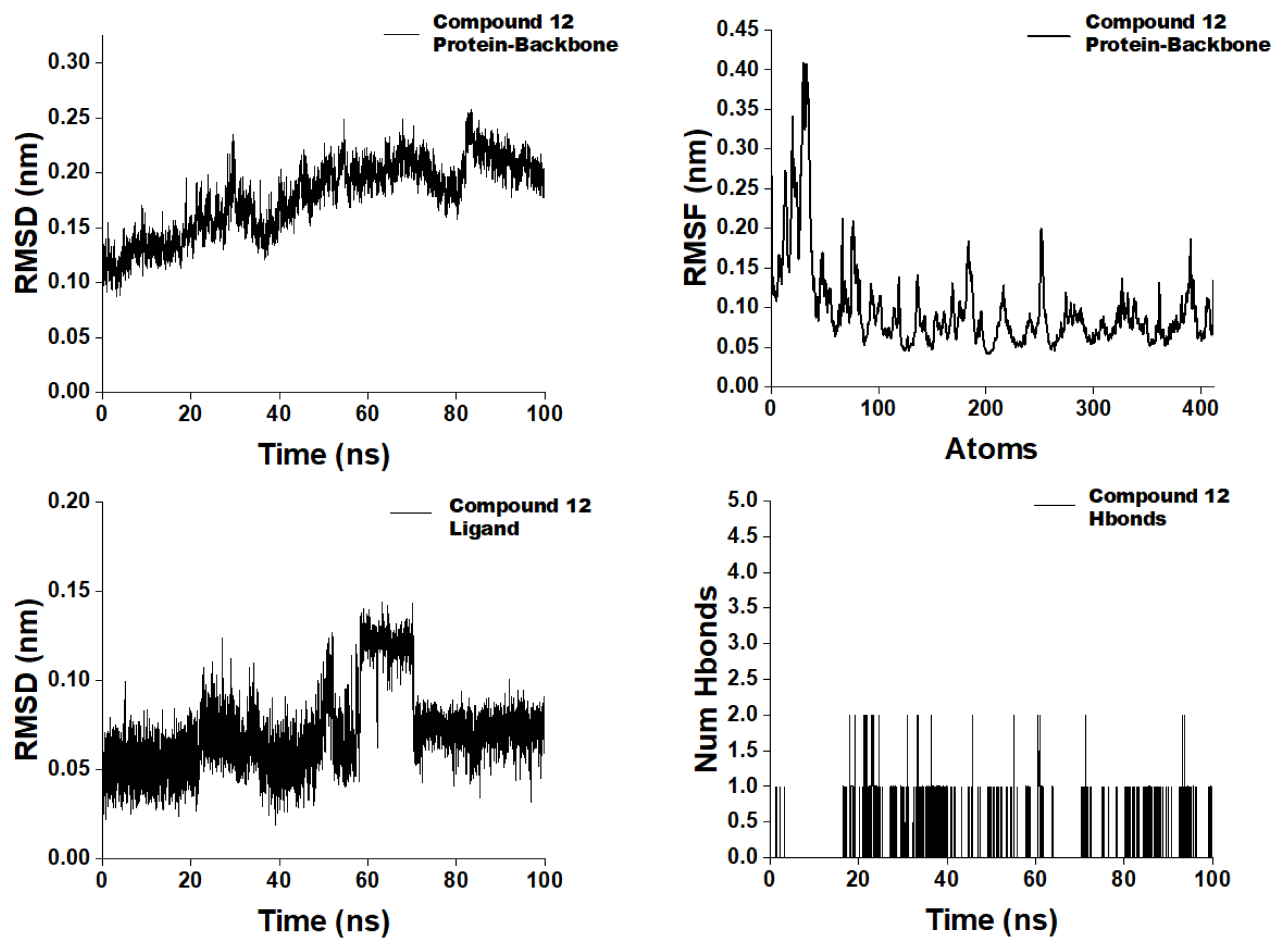
Molecular dynamics analysis of COX-2 in complex with compound **12**.

**Figure 8:**
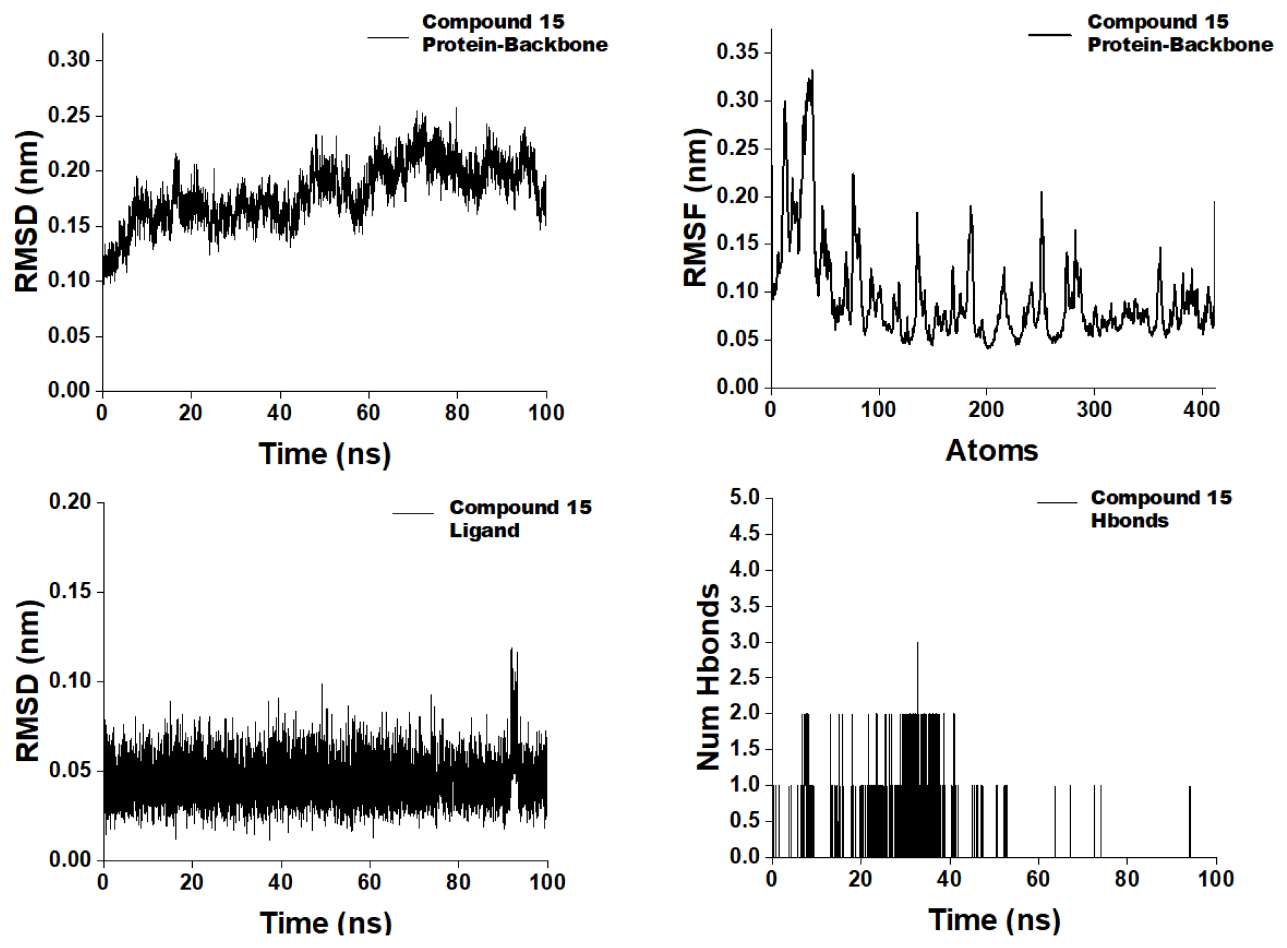
Molecular dynamics analysis of COX-2 in complex with compound **15**.

The backbone RMSD profiles indicate that all systems reached equilibrium after an initial adjustment phase, although with notable differences in stability. Compound **2** showed relatively stable behavior (Figure 5), with minor fluctuations and a short deviation around 80 ns. The COX-2–compound **8** complex demonstrated the most stable trajectory, maintaining RMSD values within a narrow range (~0.13–0.20 nm) throughout the simulation (Figure 6). In contrast, compounds **12** and **15** exhibited more pronounced fluctuations, suggesting reduced structural stability of the protein when bound to these ligands (Figure 7 and Figure 8) [24].

The ligand RMSD plots provide additional insight into binding stability (Figure 6). Compound **8** remained consistently stable within the binding pocket, showing only small positional variations. Compounds **2** and **12** displayed noticeable conformational changes during the simulation, particularly in the middle stages, indicating partial rearrangement within the active site. Although compound **15** exhibited low RMSD values, this likely reflected restricted movement rather than optimal binding interactions [25].

The RMSF analysis further revealed differences in protein flexibility. While all systems showed flexibility in loop regions, as expected, the complex with compound **8** displayed lower fluctuations in the residues surrounding the active site, indicating a stabilizing effect. In contrast, compounds **12** and **15** were associated with increased local flexibility, particularly in regions adjacent to the binding pocket [26].

Hydrogen bond analysis clearly distinguished the binding behavior of the studied compounds. Compound **8** maintained a higher and more consistent number of hydrogen bonds (typically 1–2; occasionally more) throughout the simulation, indicating strong and persistent interactions. In comparison, compounds **2** and **12** formed fewer and more intermittent hydrogen bonds, while compound **15** showed the lowest hydrogen bond occupancy, with mostly transient interactions.

Overall, the MD results confirm that compound **8** forms the most stable complex with COX-2, while compound **15** exhibits the weakest binding stability. Compounds **2** and **12** demonstrate intermediate behavior. These findings are consistent with the docking results and highlight the importance of interaction strength and stability in determining ligand efficiency within the COX-2 active site.

### 2.6. Predictive ADMET Study

A predictive ADMET study was conducted to determine the pharmacokinetic profiles and evaluate the drug-likeness of the tested compounds. The results show that their chemical structures exhibited a strong correlation with various parameters, such as aqueous solubility, absorption level, blood–brain barrier permeability, hepatotoxicity probability, atom-based log P98 (A LogP98), plasma protein binding (PPB) level, cytochrome P450 2D6 (CYP2D6), and 2D polar surface area (ADMET 2D PSA).

## 3. Discussion

The structure–activity relationship analysis showed that the highest anti-inflammatory activity was exhibited by monosubstituted derivatives in ring C containing amino, dimethylamino, nitro groups, or a bromine atom at the C-3′ or C-4′ carbon atoms (compounds **33** > **16** > **15** > **2** > **13** > **7** > **8**). Their anti-inflammatory effect (AE), in both models, ranged from 81.2% to 67.5%. The AE of these compounds exceeded that of the reference drug sodium diclofenac at doses of 8–10 mg/kg and was comparable to that of ketoprofen at a dose of 5 mg/kg. A positive influence of substituents (OH, OCH_3_, Cl, NO_2_) in the ortho position (C-2′) on AE was observed compared with substitution at C-4′ (**3** > **4**, **6** > **5**, **7** > **9**, **10** > **12**), whereas for bromine-containing compounds, the opposite trend was noted (**15** > **13** > **14**). The absence of substituents at these positions or substitution at C-2′ or C-3′ with nitro, amino, or methoxy groups led to a decrease in anti-inflammatory effect to 67–61% (**7** > **8** > **34** > **32** > **6**). Replacement of the hydrogen atom at these positions with Cl, OH, Br, or a nitro group resulted in a further reduction of AE to 52.7–44.8% (**11** > **10** > **3** > **14** > **9**).

Among disubstituted derivatives, high anti-inflammatory activity (81–71%) was demonstrated by compounds bearing methoxy and nitro, bromine and methoxy, or methylenedioxy substituents at the C-2′ and C-3′ or C-3′ and C-4′ carbon atoms (**23** > **19** > **29**). The introduction of a methoxy group at the 3′ or 4′ position of monosubstituted compounds increased their activity (**23** > **7**, **19** > **14**).

Compound **24**, containing three substituents at the C-2′, C-3′, and C-4′ positions (2-Br, 3-OH, and 4-OCH_3_), exhibited good activity (76.8%). Other derivatives with simultaneous substitution at three carbon atoms, including the C-5′ atom, showed moderate anti-inflammatory effects in the range of 52.7–29.8% (**26** > **30** > **25** > **27** > **28**). Among them, the most active were derivatives containing methoxy or methylenedioxy groups at C-4′ and C-5′ and a nitro group or bromine atom at C-2′ (compounds **26** and **30**).

Examination of the effect of 1,2,3,4-tetrahydroisoquinoline (THIQ) derivatives on the levels of anti-inflammatory and pro-inflammatory cytokines in the blood serum of rats with experimentally induced inflammation showed that many of the compounds exhibiting high anti-inflammatory activity (60% and above) modulated, to varying degrees, the levels of pro- and anti-inflammatory cytokines (Table 2), promoting a faster rapid return to values close to those of intact animals. In contrast, these parameters remained relatively elevated in the two control groups even on the seventh day of the experiment.

Data obtained using the carrageenan rat model indicate the anti-exudative activity of the compounds examined. The results obtained using the formalin rat model demonstrate these compounds’ potential antiproliferative effect.

The combined data from the in vivo study indicate that the anti-inflammatory effects of the leading substances from the examined series of 1-aryl-1,2,3,4 -tetrahydroisoquinolines may be related to their action on COX-1 and COX-2 enzymes and their regulation of cytokine levels. Unfortunately, most of the compounds, except for **5** and **19**, did not show selectivity for COX-2. Substance **5** had a moderate effect on PGE2 levels and a low effect on TXB2 levels. It did not demonstrate high AIE in the formalin rat model, while **19** was highly effective at moderating PGE2 levels but less effective at moderating TXB2 levels, showing 77% AIE in the formalin rat model. Comparisons of acute toxicity [15] and anti-inflammatory activity indicated that these compounds had relatively low toxicity (1040.3 and 964.8 mg/kg, respectively). Substance **19** (brominated in the 3′-position and with a methoxy-group in the 4′-position) may be a leading compound for the development of new selective non-steroidal anti-inflammatory drugs. Substances **9**, **12**, **17**, **22**, **27**, and **30** showed low inhibitory activity against COX-1 and COX-2. Substances **2**, **7**, **15**, **16**, **23**, **24**, **29** and **33** demonstrated good pro-inflammatory and anti-inflammatory effects, an ability to maintain cytokine balance, highly effective COX-2 inhibitions, and high or moderately effective COX-1 inhibition.

In literature, isoquinolines represent a versatile and scientifically significant group of nitrogen-containing heterocyclic compounds. Their rich chemistry, diverse biological activities, and broad range of practical applications continue to attract attention from chemists, pharmacologists, and material scientists. The study of isoquinolines not only contributes to a deeper understanding of heterocyclic chemistry but also provides valuable insights into the development of novel drugs and functional materials [27].

Ko et al. [28] compare a limited series of synthesized THIs on HO-1 induction and inhibitory effect of iNOS and COX-2 expression in lipopolysaccharide (LPS)-activated RAW264.7 cells. To the end, the most promising compound (THI-61) was tested whether this compound reduces iNOS protein expression and inflammatory markers (HMGB1, TNF-α) in LPS-treated mice lung tissue. The results indicated that N-carbonyl substituted THI seem to affect HO-1 induction depending on which functional group is attached to C1 position. All compounds that reduce LPS-activated NF-κB-luciferase activity showed to preferential inhibition of iNOS/NO but not COX-2/PGE_2_ that was partly related to inhibition of STAT-1 phosphorylation. Authors concluded that (1) lipophilic moiety of 1C substituent is much more important in N-carbonyl substituted THI for induction of HO-1, (2) newly synthesized THI-61 may be beneficial.

(S)-1-α-Naphthylmethyl-6,7-dihydroxy-1,2,3,4-tetrahydroisoquinoline (CKD712) is noted as a drug candidate for sepsis. Many studies have demonstrated its significant anti-inflammatory effects. Obtained results suggest that CKD712 inhibits LPS-induced AA release through the inhibition of a MAPKs/NF-κB pathway leading to reduced cPLA_2_ expression in RAW 264.7 cells [29].

Thirupataiah and co-workers [30] synthesized novel PDE4 inhibitors on the base of novel isoquinolin-1(2*H*)-one derivatives via a coupling–cyclization strategy.

The alkaloid 2-methoxy-4-(7-methoxy-1,2,3,4-tetrahydroisoquinolin-1-yl)phenol (MHTP) was synthesized to prospect new compounds with therapeutic properties and evaluated for anti-inflammatory effect by in vivo and in vitro assays [31]. It was founded that MHTP pre-treatment (2.5–10 mg/kg) showed antiedematogenic effect (p50.05) in carrageenan-induced paw edema by inhibiting the PGE2 action independently of mast cell degranulation or histamine activity. MHTP also diminished (p50.01) total leukocyte migration in 41.5% into peritoneal cavity during carrageenan-induced peritonitis, reducing polymorphonuclear cells (PMNs) (59.6%) and protein levels (29.4%). However, further studies will be carried out to demonstrate the mechanisms of action of the molecule and explore its potential as a future drug to treat inflammatory processes.

Therefore, our study demonstrated that the derivatives of TGIQ present anti-inflammatory activity by inhibiting some inflammatory mediators.

## 4. Materials and Methods

### 4.1. Synthesis of the Compounds

Series of derivatives of 1-aryl-1,2,3,4-tetrahydroisoquinoline were obtained as described in [15,16].

### 4.2. Biological Evaluation

The animal study was conducted according to the Institute of the Chemistry of Plant Substances, Academy of Sciences of Uzbekistan approved the protocol based on an annual working plan of the Department of Pharmacology and Toxicology (Protocol No. 1 from 16 January 2025).

The study was conducted on outbred white male rats weighing 200–220 g. The animals were maintained under standard laboratory conditions: temperature at 23 ± 2 °C, relative humidity of 48%, 12/12 h light/dark cycle, and ad libitum access to food and water.

Dose selection and administration methods were carried out in accordance with the requirements of current guidelines for preclinical studies [32,33]. The study was performed in compliance with bioethical standards, including the European Convention for the Protection of Vertebrate Animals used for Experimental and Other Scientific Purposes (Strasbourg, 1986) and EU Directive 2010/63/EU [34,35].

The anti-inflammatory effects of the tested substances were examined in white male rats weighing 180–220 g, which were divided into the following groups: intact, control (formalin model), sodium diclofenac group, ketoprofen group, and experimental groups (for checking each substance separately). A simple randomization process (coin toss considering “heads or tails”) was used for animal group formation. Each group contained 6 animals.

#### 4.2.1. Anti-Inflammatory Effects in a Formalin Rat Model

The effects of the tested compounds on the inflammatory response development were investigated using a formalin rat model, in which formalin was injected into the hind paw pads.

The levels of anti-inflammatory and pro-inflammatory cytokines were determined after subcutaneous injection of 0.1 mL of a 2% formalin solution into the pelvic region of the rats. To evaluate the dynamics of major cytokine production during the experiment, blood samples were collected from the animals before the start of the experiment and on the 7th day after its initiation. The concentrations of the principal cytokines—interleukin-4 (IL-4), interleukin-6 (IL-6), and tumor necrosis factor (TNF)—were measured in rat serum by ELISA using a Mindray MR-96A microplate immunoassay analyzer (Shenzhen Mindray Bio-Medical Electronics Company Limited, Shenzhen, China).

Data are presented as means ± SDs for different groups. Statistical analysis was performed using IBM^®^ SPSS^®^ Statistics v27.0.1.0 software, and significant differences between groups were assessed using Student’s *t*-test.

#### 4.2.2. Anti-Inflammatory Effects in a Carrageenan Rat Model

Six animals were randomly selected to be allocated to one of the following groups: an intact group, a control group (physiological solution, 0.1 mL/100 g body weight), a group administered the reference drug—Dikloberl retard (8.0–10.0 mg/kg, Berlin-Chemie AG Menarini Group, Berlin, Germany)—and experimental groups administered the 33 test substances (at doses of 1.0–5.0–10.0 mg/kg).

At the beginning of the experiment, the normal size of the right hind paw of the animals was measured. The reference drug and test substances were administered orally (intragastrically) using an atraumatic probe.

Then, 100 μL of 1% λ-carrageenan solution (Sigma-Aldrich, St. Louis, MO, USA) was injected subcutaneously into the right hind paw of the experimental animals. Paw volume was determined using a plethysmometer (Ugo Basile, Gemonio, Italy) at 60, 120, and 180 min after carrageenan injection.

Blood samples were taken from the experimental animals 240 min after carrageenan administration. Plasma was separated in a centrifuge (SIA Biosan Ratsupites iela 7 k-2, Riga, Latvia, LV-1067) at 1200 rpm for 10 min.

The levels of prostaglandin E_2_ (PGE_2_) and thromboxane B_2_ (TXB_2_) in plasma were determined using commercially available competitive ELISA kits (Arbor Assays, Ann Arbor, MI, USA) on an Accuris Smartreader ^TM^ 96 (Benchmark Scientific, Inc., Sayreville, NJ, USA) immunoenzyme analyzer.

### 4.3. Molecular Docking

Among the three known COX isoforms (COX-1, COX-2, and COX-3), the inducible enzyme COX-2 is recognized as the most active during inflammatory processes [32]. Molecular docking studies of the 34 tested compounds were conducted against the cyclooxygenase-2 (COX-2) enzyme (PDB ID: 1PXX), with the crystal structure obtained from the Worldwide PDB Protein Data Bank [36,37,38,39]. For the simulation, the A chain of the protein was specifically selected.

Ligand–receptor interactions were modeled using the AutoDock 4.0 software within the MGL Tools 1.5.6 suite, employing the Lamarckian Genetic Algorithm (LGA) [30]. Preliminary protein preparation involved the removal of all water molecules from the crystal structure. Receptor and ligand files were converted to PDBQT format, incorporating missing hydrogen atoms and assigning partial atomic charges via the Gasteiger method. The grid box dimensions were set at 40 × 40 × 40 Å with the central coordinates x = 29.51, y = 22.92, and z = 17.87, encompassing the active binding site.

Visual inspection of the docking results and analysis of the interaction energy (E) were further performed using Discovery Studio 4.0 [40]. This facilitated a comparative analysis of the binding modes of the tested compounds against reference ligands and reported literature. The best-scoring pose for each compound was selected to represent key interactions with essential amino acid residues, including hydrogen bonding, hydrophobic interactions (pi–pi, pi–alkyl), and Van der Waals forces. Additionally, a predictive in silico ADMET study was conducted using Discovery Studio 4.0 to evaluate the pharmacokinetic profiles and drug-likeness of the compounds, as well as to screen for potential toxicity [41].

### 4.4. Molecular Dynamics Simulation

Molecular dynamics (MD) simulations were performed to evaluate the stability of the selected ligand–protein complexes obtained from docking analysis. Compounds **2**, **8**, **12**, and **15**, which showed favorable docking scores and interaction profiles with COX-2 (PDB ID: 1PXX), were chosen for further investigation [40].

All simulations were carried out using the GROMACS 2023.3 package. The protein was described using the AMBER99SB-ILDN force field, while ligand topologies were generated using the General AMBER Force Field (GAFF) via ACPYPE. The ligand charges were assigned using the AM1-BCC method [41].

Each protein–ligand complex was placed in a cubic simulation box and solvated with the TIP3P water model. Counter ions were added to neutralize the system. Energy minimization was performed using the steepest descent algorithm until convergence.

Following minimization, the system was equilibrated in two steps: the first step was under NVT conditions for 100 ps to stabilize temperature at 300 K using the V-rescale thermostat, which was followed by NPT equilibration for 100 ps at 1 bar using the Parrinello–Rahman barostat.

Production MD simulations were then performed for 100 ns under periodic boundary conditions. The LINCS algorithm was applied to constrain all bond lengths, and a time step of 2 fs was used. Long-range electrostatic interactions were calculated using the Particle Mesh Ewald (PME) method, with a cutoff of 1.0 nm applied for both electrostatic and van der Waals interactions.

Trajectory analysis was carried out using standard GROMACS tools to calculate the root mean square deviation (RMSD), root mean square fluctuation (RMSF), ligand RMSD, and hydrogen bond interactions throughout the simulation.

### 4.5. Predictive ADMET Study

A predictive ADMET study was conducted using Discovery Studio 4.0 software to determine the pharmacokinetic profiles and evaluate the drug-likeness of the tested compounds.

## Figures and Tables

**Table 1:** Anti-inflammatory effects of 1-aryl-1,2,3,4-tetrahydroisoquinolines at a dose of 1 mg/kg (p.o.) in a formalin rat model.

Examined Compound (Substituent)	AIE, %	Examined Compound (Substituent)	AIE, %
Control (formalin)	-	Ketoprofen (5 mg/kg)	57.6
		Sodium diclofenac (8 mg/kg)	66.3
1-Phenyl- (**2**)	70.1	1-(3′-Bromo-4′-hydroxyphenyl)- (**18**)	52.0
1-(2′-Hydroxyphenyl)- (**3**)	46.8	1-(3′-Bromo-4′-methoxyphenyl)- (**19**)	76.8
1-(4′-Hydroxyphenyl)- (**4**)	23.1	1-(3′-Hydroxy-4′-methoxyphenyl)- (**20**)	54.1
1-(4′-Methoxyphenyl)- (**5**)	27.5	1-(4′-Hydroxy-3′-methoxyphenyl)- (**21**)	50.7
1-(2′-Methoxyphenyl)- (**6**)	61.3	1-(3′,4′-Dimethoxyphenyl)- (**22**)	57.6
1-(2′-Nitrophenyl)- (**7**)	69.3	1-(3′-Methoxy-2′-nitrophenyl)- (**23**)	80.8
1-(3′-Nitrophenyl) (**8**)	67.5	1-(2′-Bromo-5′-hydroxy-4′-methoxyphenyl)- (**24**)	76.8
1-(4′-Nitrophenyl)- (**9**)	44.8	1-(2′-Bromo-4′,5′-dimethoxy)- (**25**)	42.3
1-(2′-Chlorophenyl)- (**10**)	50.8	1-(4′,5′-Dimethoxy-2′-nitrophenyl)- (**26**)	52.7
1-(3′-Chlorophenyl)- (**11**)	52.7	1-(2′-Bromo-3′-hydroxy-4′-methoxy)- (**27**)	34.9
1-(4′-Chlorophenyl)- (**12**)	40.1	1-(5′-Bromo-4′-hydroxy-3′-methoxyphenyl)- (**28**)	29.8
1-(2′-Bromophenyl)- (**13**)	69.5	1-(3′,4′-Methylenedioxyphenyl)- (**29**)	71.1
1-(3′-Bromophenyl)- (**14**)	44.6	1-(2′-Bromo-4′,5′-Methylenedioxyphenyl)- (**30**)	46.8
1-(4′-Bromophenyl)- (**15**)	77.1	1-(4′,5′-Methylenedioxy-2′-chlorophenyl)- (**31**)	40.6
1-(4′-Dimethylaminophenyl)- (**16**)	77.6	1-(2′-Aminophenyl)- (**32**)	63.9
1-(5′-Bromo-2′-hydroxyphenyl)- (**17**)	53.5	1-(3′-Aminophenyl)- (**33**)	81.2
		1-(4′-Aminophenyl)- (**34**)	66.0

Data are shown as mean ± SD of different groups (N = 6). *p* < 0.05 is considered statistically significant.

**Table 2:** Effects of the examined substances on the cytokine levels in blood serum of rats at a dose of 1 mg/kg (p.o.) in a formalin rat model.

Examined Compound	Dose, mg/kg	IL-4, pg/mL	IL-6, pg/mL	TNF, pg/mL
Control 1 (intact animals)		0.16 (0.145–0.175)	0.31 (0.29–0.33)	0.65 (0.32–0.68)
Control 2 (formalin)		2.0 (1.84–2.16) *	2.4 (1.37–3.43) *	1.275 (2.93–3.26) *
**2**	1.0	0.22 (0.150–0.178) **	0.09 (0.02–0.15) **	0.97 (0.88–1.06) **
**7**	1.0	0.40 (0.166–0.190) **	0.13 (0.10–0.17) **	1.15 (1.06–1.25) **
**15**	1.0	0.44 (0.141–0.148) **	0.07 (0.02–0.11) **	0.97 (0.90–1.04) **
**16**	1.0	0.41 (0.168–0.192) **	0.07 (0.03–0.11) **	0.97 (0.90–1.04) **
**19**	1.0	0.45 (0.171–0.192) **	0.09 (0.07–0.12) **	0.97 (0.93–1.00) **
**23**	1.0	0.42 (0.164–0.196) **	0.06 (0.03–0.08) **	0.92 (0.88–0.96) **
**24**	1.0	0.34 (0.165–0.190) **	0.08 (0.05–0.11) **	0.98 (0.94–1.02) **
**29**	1.0	0.35 (0.164–0.188) **	0.07 (0.04–0.09) **	0.96 (0.92–1.00) **
**33**	1.0	0.36 (0.169–0.190) **	0.07 (0.04–0.10) **	0.94 (0.90–0.98) **

* reliability relative to intact group; ** reliability relative to control group (*p* < 0.05).

**Table 3:** Effects of the examined compounds on plasma PGE_2_ levels in a carrageenan-induced inflammation model (M ± SEM, n = 6).

Examined Compound	Dose, mg/kg	PGE_2_ Level, pg/mL	Examined Compound	Dose, mg/kg	PGE_2_ Level, pg/mL
Intact		352.6 ± 15.0	**17**	1.0	654.9 ± 20.0 *
Control carrageenan 1.0% 0.1 mL	0.2 mL	967.2 ± 25.0	**18**	1.0	524.5 ± 19.5 **
Dicloberl retard + carrageenan 1.0% 100 μL	8.0	536.5 ± 20.0 *	**19**	1.0	475.5 ± 17.0 **
	10.0	511.3 ± 19.0 *			
**2**	1.0	495.4 ± 18.0 **	**20**	1.0	502.2 ± 18.5 **
**3**	1.0	561.5 ± 21.0 **	**21**	1.0	562.5 ± 21.0 **
**4**	1.0	588.7 ± 22.0 **	**22**	1.0	638.4 ± 23.5 *
**5**	1.0	624.2 ± 23.0 *	**23**	1.0	459.4 ± 17.0 **
**6**	1.0	546.3 ± 20.0 **	**24**	1.0	537.0 ± 20.0 **
**7**	1.0	518.6 ± 19.0 **	**25**	1.0	582.2 ± 21.5 **
**8**	1.0	503.1 ± 18.5 **	**26**	1.0	532.7 ± 19.5 **
**9**	1.0	639.9 ± 24.0 *	**27**	1.0	672.1 ± 24.5 *
**10**	1.0	487.1 ± 18.0 **	**28**	1.0	644.4 ± 24.0 *
**11**	1.0	582.7 ± 21.5 **	**29**	1.0	498.6 ± 18.5 **
**12**	1.0	705.1 ± 26.0 *	**30**	1.0	649.3 ± 24.0 *
**13**	1.0	502.9 ± 18.5 **	**31**	1.0	525.9 ± 19.5 **
**14**	1.0	547.3 ± 20.0 **	**32**	1.0	502.3 ± 18.5 **
**15**	1.0	481.4 ± 17.5 **	**33**	1.0	451.4 ± 16.5 **
**16**	1.0	512.5 ± 19.0 **	**34**	1.0	479.9 ± 17.5 **

Note: Data are presented as mean ± SD (n = 6). * *p* < 0.05; ** *p* < 0.01—significant difference compared to the carrageenan control group. Intact—physiological group, control—1% λ-carrageenan. The reference drug is sodium diclofenac (Dikloberl retard).

**Table 4:** Effects of the examined compounds on plasma TXB_2_ levels in a carrageenan-induced inflammation model (M ± SEM, n = 6).

Examined Compound	Dose, mg/kg	Decrease in TXB_2_ Level, %	Examined Compound	Dose, mg/kg	Decrease in TXB_2_ Level, %
Intact		100 174 52.1 *	**18**	1.0	45.3 *
Control carrageenan 1.0% 0.1 mL	0.2 mL	45.0 ** 40.4 ** 37.1 **	**19**	1.0	85.7
Dicloberl retard + carrageenan 1.0% 100 μL	8.0	54.3 *	**20**	1.0	48.7 *
	10.0	59.2 * 55.2 *			
**2**	1.0	53.7 *	**21**	1.0	34.8 **
**3**	1.0	64.4	**22**	1.0	42.4 **
**4**	1.0	67.6	**23**	1.0	59.5 *
**5**	1.0	35.0 **	**24**	1.0	81.0
**6**	1.0	30.9 **	**25**	1.0	30.2 **
**7**	1.0	32.8 **	**26**	1.0	52.2 *
**8**	1.0	98.1	**27**	1.0	65.1
**9**	1.0	30.3 **	**28**	1.0	51.0 *
**10**	1.0	37.4 **	**29**	1.0	90.6
**11**	1.0	92.4	**30**	1.0	82.7
**12**	1.0	38.1 **	**31**	1.0	41.4 **
**13**	1.0	65.2	**32**	1.0	84.1
**14**	1.0	99.9	**33**	1.0	49.1 *
**15**	1.0	42.6 **	**34**	1.0	42.4 **
**16**	1.0	55.2 *	**35**	1.0	28.0 **
**17**	1.0	36.5 **	**36**	1.0	36.1 **

Note: Data are presented as mean ± SD (n = 6). * *p* < 0.05; ** *p* < 0.01—significant difference compared to the carrageenan control group. Intact—physiological group, control—1% λ-carrageenan. The reference drug is sodium diclofenac (Dikloberl retard).

**Table 5:** Docking score (Kcal/mol) and interactions of compounds **2**–**34** at the active site of COX-2 (PDB ID: 1PXX).

Compound	Ligand/C-Docker Interaction Energy (Kcal/mol)	Amino Acid Residue Interactions
Ligand (2-[2-(2,6-dichloroanilino)-phenyl] acetic acid)	−7.90	1 HBD with **SER530**, 1 pi-sulfur bond with **MET522**, 1 pi-pi t-shaped bond with **TRP387**, 1 pi–alkyl bond with **VAL349**, **VAL523**, and **ALA527**
**2**	−7.83	1 carbon–hydrogen bond with **GLY526**, 1 carbon–hydrogen bond and 1 alkyl bond with **MET522**, 1 alkyl bond with **LEU384**, 1 pi–alkyl bond with **PHE381**, **TYR385**, **PHE518**, **LEU352**, and **ALA527** and **ALA516**, 2 pi–alkyl bonds with **TRP387** and **VAL523**
**3**	−8.06	1 HBD and 1 pi–alkyl bond with **TYR355**, 1 HBA and 1 pi-sulfur bond with **MET522**, 1 pi-pi t-shaped bond with **TRP387**, 1 alkyl bond and 1 pi–alkyl bond with **ALA527**, **VAL523**, and **VAL349**, 1 alkyl bond with **LEU531**
**4**	−7.94	1 HBD and 1 pi–alkyl bond with **PHE518**, 1 HBA with **GLN192**, 1 carbon–hydrogen bond with **GLY526**, 1 carbon–hydrogen bond and 1 alkyl bond with **MET522**, 1 pi-long pair and 2 pi–alkyl bonds with **LEU352**, 1 alkyl bond and 1 pi–alkyl bond with **ALA527**, 1 alkyl bond with **VAL34** and **LEU384**, 1 alkyl bond and 2 pi–alkyl bonds with **VAL523**, 1 pi–alkyl bond with **PHE381**, **TYR385**, **TRP387**, and **ALA516**
**5**	−7.87	1 HBA and 1 carbon–hydrogen bond with **MET522**, 3 carbon–hydrogen bonds with **SER353**, 1 carbon–hydrogen bond with **SER530**, 1 amide–pi stacked bond with **GLY526**, 1 alkyl bond with **LEU384** and **VAL349**, 1 alkyl bond and 1 pi–alkyl bond with **VAL523**, 1 pi–alkyl bond with **HIS90**, **TYR355**, **PHE205**, **PHE381**, **TRP387**, and **ALA527**, 2 bonds with **TYR385**
**6**	−8.05	1 HBD with **ARG120**, 1HBD and 1 pi–alkyl bond with **TYR355**, 2 carbon–hydrogen bonds and 1 alkyl bond with **MET522**, 1 alkyl bond and 1 pi–alkyl bond with **VAL523**, **VAL349**, and **ALA527**, 1 alkyl bond with **LEU384** and **LEU531**, 2 pi–alkyl bonds with **TRP387**, 1 pi–alkyl bond with **PHE518** and **LEU352**
**7**	−9.16	1 HBD with **ARG120** and **SER530**, 1 HBD and 1 pi–alkyl bond with **TYR355**, 1 carbon–hydrogen bond with **LEU352**, 1 pi-sulfur bond with **MET522**, 1 pi-pi t-shaped bond with **TRP387**, 1 alkyl bond and 1 pi–alkyl bond with **VAL523**, **VAL349**, and **ALA527**, 1 pi–alkyl bond with **LEU531**
**8**	−9.66	2 HBDs with **ARG120**, 1 carbon–hydrogen bond and 1 alkyl bond with **MET522**, 1 amide–pi stacked bond with **GLY526**, 1 carbon–hydrogen bond with **TYR385**, 1 alkyl bond with **LEU384**, **VAL349**, and **VAL344**, 1 pi–alkyl bond with **PHE205**, **TYR348**, **TRP387**, **VAL523**, and **ALA527**, 2 pi–alkyl bonds with **TYR385**
**9**	−8.69	1 HBD with **HIS90**, **ILE517**, 1 HBD and 1 pi–alkyl bond with **PHE518**, 1 carbon–hydrogen bond with **ARG513** and **GLY526**, 1 carbon–hydrogen bond and 1 alkyl bond with **MET522**, 1 alkyl bond and 1 pi–alkyl bond with **ALA527**, 1 alkyl bond with **VAL349** and **LEU384**, 1 alkyl bond and 2 pi–alkyl bonds with **VAL523**, 1 pi–alkyl bond with **PHE381**, **TYR385**, and **LEU352**, 2 pi–alkyl bonds with **TRP387**
**10**	−8.37	1 HBD and 1 pi–alkyl bond with **TYR355**, 1 pi-sulfur bond with **MET522**, 1 pi-pi t-shaped bond with **TRP387**, 1 alkyl bond and 1 pi–alkyl bond with **ALA527**, **VAL523**, and **VAL349**, 1 alkyl bond with **LEU531**
**11**	−8.36	1 HBD with **SER530**, 1 carbon–hydrogen bond and 1 alkyl bond with **MET522**, 1 carbon–hydrogen bond and 2 pi–alkyl bonds with **TYR385**, 1 amide–pi stacked bond with **GLY526**, 1 alkyl bond with **LEU384** and **VAL349**, 1 alkyl bond **(halogen bond)** and 1 pi–alkyl bond with **VAL523**, 1 pi–alkyl bond with **PHE205**, **TYR348**, **TYR355 (halogen bond)**, and **LEU352**, 2 pi–alkyl bonds with **TRP387**
**12**	−8.14	1 carbon–hydrogen bond with **GLY526**, 1 carbon–hydrogen bond and 1 alkyl bond with **MET522**, 1 alkyl bond with **ALA516 (halogen bond)**, **ARG513 (halogen bond)**, **VAL349**, and **LEU384**, 1 alkyl bond and 1 pi–alkyl bond with **ALA527**, 1 pi–alkyl bond with **HIS90 (halogen bond)**, **PHE381**, **TYR385**, **PHE518**, and **LEU352**, 2 pi–alkyl bonds with **TRP387** and **VAL523**
**13**	−8.38	1 HBD and 1 pi–alkyl bond with **TYR355**, 1 pi-donor hydrogen bond with **SER530**, 1 alkyl bond with **LEU531**, 1 alkyl bond and 1 pi–alkyl bond with **ALA527** and **VAL523**, 1 alkyl bond and 2 pi–alkyl bonds with **VAL349**, 1 pi–alkyl bond with **PHE205**, **TYR385**, and **LEU352**
**14**	−8.57	1 HBD and 1 pi–alkyl bond with **TYR355**, 1 pi-sulfur bond with **MET522**, 1 pi-pi t-shaped bond with **TRP387**, 1 alkyl bond with **LEU531**, 1 alkyl bond and 1 pi–alkyl bond with **ALA527**, **VAL523**, and **VAL349**, 1 pi–alkyl bond with **PHE381 (halogen bond)**, **TYR385 (halogen bond)**, and **LEU352**
**15**	−8.26	1 carbon–hydrogen bond with **GLY526**, 1 carbon–hydrogen bond and 1 alkyl bond with **MET522**, 1 carbon–hydrogen bond and 1 pi–alkyl bond with **TYR385**, 1 pi-sigma bond with **SER353**, 1 alkyl bond with **ALA516 (halogen bond)**, **VAL349**, **ARG513 (halogen bond)**, and **LEU384**, 1 alkyl bond and 1 pi–alkyl bond with **ALA527**, 1 alkyl bond **(halogen bond)** and 2 pi–alkyl bonds with **VAL523**, 1 pi–alkyl bond with **HIS90 (halogen bond)**, **PHE381**, **TRP387**, **PHE518**, and **LEU352**
**16**	−8.25	1 carbon–hydrogen bond with **GLY526** and **GLN192**, 1 carbon–hydrogen bond and 1 alkyl bond with **PHE518**, 1 carbon–hydrogen bond and 1 alkyl bond with **MET522**, 1 carbon–hydrogen bond and 1 pi-sigma bond with **SER353**, 1 alkyl bonds with **VAL349**, **ARG513**, **LEU384**, 2 alkyl bonds with **ALA516**, 1 alkyl bond and 1 pi–alkyl with **ALA527**, 1 alkyl bond and 2 pi–alkyl bonds with **VAL523**, 1 pi–alkyl bond with **PHE381**, **TYR385**, **TRP387**, and **LEU352**, 2 pi–alkyl bonds with **HIS90**
**17**	−8.54	1 HBA with **MET522**, 1 HBD and 1 pi–alkyl bond with **TYR355**, 1 pi-pi t-shaped bond with **TRP387**, 1 alkyl bond with **LEU531**, 1 alkyl bond and 1 pi–alkyl bond with **ALA527**, **VAL523**, and **VAL349**, 1 pi–alkyl bond **(halogen bond)** with **PHE381** and **TYR385**
**18**	−8.48	1 HBA and 1 pi–alkyl bond with **TYR385 (halogen bond)**, 1 HBD and 1 pi–alkyl bond with **TYR355**, 1 alkyl bond with **LEU384 (halogen bond)**, **LEU531**, 1 alkyl bond and 1 pi–alkyl bond with **ALA527** and **VAL523**, 1 alkyl bond and 2 pi–alkyl bonds with **VAL349**, 1 pi–alkyl bond with **PHE381 (halogen bond)**, **TRP387 (halogen bond)**, and **LEU352**
**19**	−8.48	1 carbon–hydrogen bond with **SER353**, 1 amide–pi stacked bond with **GLY526**, 1 alkyl bond with **LEU384** and **VAL344**, 2 alkyl bonds with **VAL349**, 2 alkyl bonds and 1 pi–alkyl bond with **VAL523 (halogen bond)**, 1 pi–alkyl bond with **HIS90**, **PHE205**, **TYR348**, **TYR355**, **TYR385**, **PHE518 (halogen bond)**, and **ALA527**, 2 pi–alkyl bonds with **TRP387**
**20**	−8.35	1 HBA with **GLN192**, 1 HBD and 1 pi–alkyl bond with **HIS90**, 1 carbon–hydrogen bond with **GLY526**, 1 carbon–hydrogen bond and 1 alkyl bond with **MET522**, 1 alkyl bond with **ALA516**, **VAL349**, **ARG513**, and **LEU384**, 1 alkyl bond and 1 pi–alkyl bond with **ALA527**, 1 alkyl bond and 2 pi–alkyl bonds with **VAL523**, 1 pi–alkyl bond with **PHE381**, **TYR385**, **PHE518**, and **LEU352**, 2 pi–alkyl bonds with **TRP387**
**21**	−7.84	1 HBD and 1 pi–alkyl bond with **TYR355**, 1 pi-donor hydrogen bond with **SER530**, 1 alkyl bond with **VAL344**, **LEU534**, and **LEU531**, 1 alkyl bond and 1 pi–alkyl bond with **ALA527**, and **VAL523**, 2 alkyl bonds and 2 pi–alkyl bonds with **VAL349**, 1 pi–alkyl bond with **PHE205**, **TYR348**, and **LEU352**
**22**	−8.33	1 HBD and 2 pi–alkyl bonds with **TYR355**, 1 carbon–hydrogen bond with **SER353**, 1 amide–pi stacked bond with **GLY526**, 1 alkyl bond with **LEU384** and **VAL344**, 3 alkyl bonds with **VAL349**, 1 alkyl bond and 1 pi–alkyl bond with **ALA527** and **VAL523**, 1 pi–alkyl bond with **HIS90**, **PHE205**, **TYR348**, **TYR385**, and **PHE518**, 2 pi–alkyl bonds with **TRP387**
**23**	−8.80	1 HBD and 1 carbon–hydrogen bond with **SER530**, 1 HBD and 1 pi–alkyl bond with **TYR355**, 1 carbon–hydrogen bond and 1 pi–alkyl bond with **TYR385**, 1 pi-sulfur bond with **MET522**, 1 pi-pi t-shaped bond with **TRP387**, 1 alkyl bond with **LEU531**, 1 alkyl bond and 1 pi–alkyl bond with **ALA527**, **VAL523**, and **VAL349**, 1 pi–alkyl bond with **PHE381**
**24**	−8.37	1 HBD and 1 pi–alkyl bond with **TYR355**, 1 pi-donor hydrogen bond with **SER530**, 1 alkyl bond with **LEU531**, 1 alkyl bond and 1 pi–alkyl bond with **ALA527** and **VAL523**, 1 alkyl bond and 2 pi–alkyl bonds with **VAL349**, 1 pi–alkyl bond with **PHE205**, **PHE381**, and **TYR385**
**25**	−8.64	1 HBD and 1 pi–alkyl bond with **TYR355**, 1 pi-donor hydrogen bond with **SER530**, 1 alkyl bond with **LEU531**, **LEU534**, and **VAL344**, 1 alkyl bond and 1 pi–alkyl bond with **ALA527**, and **VAL523**, 2 alkyl bonds and 2 pi–alkyl bonds with **VAL349**, 1 pi–alkyl bond with **PHE205**, **PHE381**, **TYR348**, **TYR385**, and **LEU352**
**26**	−8.23	1 carbon–hydrogen bond with **LEU352**, 1 carbon–hydrogen bond and 1 pi–alkyl bond with **TYR355**, 1 alkyl bond with **LEU384** and **MET522**, 1 alkyl bond and 1 pi–alkyl bond with **ALA527** and **VAL349**, 2 alkyl bonds and 1 pi–alkyl bond with **VAL523**, 1 pi–alkyl bond with **HIS90**, **PHE381**, and **TYR385**, 2 pi–alkyl bonds with **TRP387** and **PHE518**
**27**	−8.24	1 HBD and 1 pi–alkyl bond with **TYR355**, 1 halogen bond and 1 alkyl bond with **MET522**, 1 alkyl bond with **LEU531** and **VAL344**, 1 alkyl bond and 1 pi–alkyl bond with **ALA527**, 1 alkyl bond and 2 pi–alkyl bonds with **VAL349**, 1 pi–alkyl bond with **PHE205**, **TYR348**, **TYR385**, **VAL523**, and **LEU352**
**28**	−8.42	1 HBD with **SER530**, 1 HBA, 1 carbon–hydrogen bond, and 1 pi–alkyl bond **(halogen bond)** with **TYR385**, 1 amide–pi stacked bond with **GLY526**, 1 alkyl bond with **LEU384 (halogen bond)**, **VAL344**, **LEU531**, and **VAL116**, 1 alkyl bond and 1 pi–alkyl bond with **ALA527**, and **VAL523**, 2 alkyl bonds and 2 pi–alkyl bonds with **VAL349**, 1 pi–alkyl bond with **TYR348**, **TYR385**, and **LEU352**, 2 pi–alkyl bonds **(halogen bond)** with **TRP387**
**29**	−8.30	1 HBD with **HIS90**, 1 carbon–hydrogen bond with **GLY526** and **GLN192**, 1 carbon–hydrogen bond and 1 alkyl bond with **MET522**, 1 pi-sigma bond with **SER353**, 1 alkyl bond with **ALA516**, **VAL349**, and **LEU384**, 1 alkyl bond and 1 pi–alkyl bond with **ALA527**, 1 pi–alkyl bond with **PHE381**, **TYR385**, and **LEU352**, 2 pi–alkyl bonds with **TRP387**, **PHE518**, and **VAL523**
**30**	−8.83	1 HBD and 1 pi–alkyl bond with **TYR355**, 1 pi-donor hydrogen bond with **SER530**, 1 alkyl bond with **LEU531**, 1 alkyl bond and 1 pi–alkyl bond with **ALA527** and **VAL523**, 1 alkyl bond and 2 pi–alkyl bonds with **VAL349**, 1 pi–alkyl bond with **PHE205**, **TYR348**, **TYR385**, and **LEU352**
**31**	−8.47	1 HBD and 1 pi–alkyl bond with **TYR355**, 1 carbon–hydrogen bond and 1 pi–alkyl bond with **TYR385**, 1 alkyl bond with **LEU531**, 1 alkyl bond and 1 pi–alkyl bond with **ALA527** and **VAL523**, 1 alkyl bond and 2 pi–alkyl bonds with **VAL349**, 1 pi–alkyl bond with **PHE381** and **LEU352**
**32**	−8.17	1 HBD and 1 pi–alkyl bond with **TYR355**, 1 HBA with **MET522**, 1 pi-pi t-shaped bond with **TRP387**, 1 alkyl bond with **LEU531**, 1 alkyl bond and 1 pi–alkyl bond with **ALA527**, **VAL523**, and **VAL349**
**33**	−8.95	1 HBD and 1 pi–alkyl bond with **TYR355**, 1 pi-pi t-shaped bond with **TRP387**, 1 alkyl bond with **LEU531**, 1 alkyl bond and 1 pi–alkyl bond with **ALA527**, **VAL523**, and **VAL349**
**34**	−8.01	1 HBD and 1 pi–alkyl bond with **TYR355**, 1 pi-donor hydrogen bond with **SER530**, 1 alkyl bond with **LEU531**, 1 alkyl bond and 1 pi–alkyl bond with **ALA527** and **VAL523**, 1 alkyl bond and 2 pi–alkyl bonds with **VAL349**

## Data Availability

The original contributions presented in this study are included in the article/Supplementary Material. Further inquiries can be directed to the corresponding author.

## Supplementary Materials

The following supporting information can be downloaded at: https://www.mdpi.com/article/10.3390/molecules31111956/s1, Table S1: Anti-inflammatory effects of 1-aryl-1,2,3,4-tetrahydroisoquinolines at three doses in a formalin rat model.; Table S2: Effects of the examined compounds at three doses on plasma PGE_2_ levels in a carrageenan-induced inflammation model; Table S3: Effects of the examined compounds at three doses on plasma TXB_2_ levels in a carrageenan-induced inflammation model.

## Abbreviations

The following abbreviations are used in this manuscript:

1-ArTHIQ	1-aryl-6,7-dimethoxy-1,2,3,4-tetrahydroisoquinoline
COX-2	Cyclooxigenase-2
