# Supplementary material for: Anti-Inflammatory Potential of 1-Aryl-6,7-Dimethoxy-1,2,3,4-Tetrahydroisoquinolines: Structure–Activity Relationship and COX-2 Binding
Source: Molecules. 2026 Jun 4;31(11):1956. doi: 10.3390/molecules31111956 (PMC13257858; doi:10.3390/molecules31111956)
Supplement: Supplementary file 1 [file molecules-31-01956-s001.zip › molecules-4223647-supplementary.pdf]

Table S1. Anti-inflammatory effects of 1-aryl-1,2,3,4-tetrahydroisoquinolines at three doses (p.o.) in a formalin rat model.

Examined Compound (Substituent)	**Dose mg/kg**	AIE, %	Examined Compound (Substituent)	**Dose mg/kg**	AIE, %
**Control (formalin)**	02 mL NS	-	1-(5′-Bromo-2′-hydroxyphenyl)- (**17**)	1.0	**53.5**
				5.0	**59.4**
				10.0	**62.7**
**Ketoprofen**	1.0	**30.4**	1-(3′-Bromo-4′-hydroxyphenyl)- (**18**)	1.0	**52.0**
	5.0	**57.6**		5.0	**70.2**
	10.0	**73.4**		10.0	**55.1**
**Sodium diclofenac**	8.0	**64.8**	**1-(3′-Bromo-4′-methoxyphenyl)- (19)**	1.0	**76.8**
	10.0	**66.3**		5.0	**69.0**
**1-Phenyl-** (2)	1.0	**70.1**		10.0	**64.0**
	5.0	**72.5**	1-(3′-Hydroxy-4′-methoxyphenyl)- (**20**)	1.0	**54.1**
	10.0	**46.8**		5.0	**76.7**
**1-(2′-Hydroxyphenyl)-** (3)	1.0	**46.8**		10.0	**69.3**
	5.0	**60.6**	1-(4′-Hydroxy-3′-methoxyphenyl)- (**21**)	1.0	**50.7**
	10.0	**68.1**		5.0	**58.2**
**1-(4′- Hydroxyphenyl)-** (4)	1.0	**23.1**		10.0	**76.8**
	5.0	**65.2**	1-(3′,4′-Dimethoxyphenyl)- (**22**)	1.0	**57.6**
	10.0	**45.7**		5.0	**55.7**
**1-(4′- Methoxyphenyl) -** (5)	1.0	**27.5**		10.0	**51.2**
	5.0	**49.9**	**1-(3′-Methoxy-2′-nitrophenyl)- (23)**	1.0	**80.8**
	10.0	**65.0**		5.0	**74.6**
**1-(2′- Methoxyphenyl)-** (6)	1.0	**61.3**		10.0	**57.3**
	5.0	**66.7**	1-(2′-Bromo-5′-hydroxy-4′-methoxyphenyl)- (**24**)	1.0	**76.8**
	10.0	**72.0**		5.0	**72.8**
1-(2′-Nitrophenyl)- (7)	1.0	**69.3**		10.0	**66.3**
	5.0	**71.3**	1-(2′-Bromo-4′,5′-dimethoxy)- (**25**)	1.0	**42.3**
	10.0	**82.3**		5.0	**52.0**
**1-(3′- Nitrophenyl)** (8)	1.0	**67.5**		10.0	**70.7**
	5.0	**70.7**	1-(4′,5′-Dimethoxy-2′-nitrophenyl)- (**26**)	1.0	**52.7**
	10.0	**80.8**		5.0	**65.6**
	1.0	**44.8**		10.0	**78.0**
**1-(4′- Nitrophenyl)-** (9)	5.0	**35.1**	1-(2′-Bromo-3′-hydroxy-4′-methoxy)- (**27**)	1.0	**34.9**
	10.0	**32.7**		5.0	**40.1**
**1-(2′-Chlorophenyl)-** (10)	1.0	**50.8**		10.0	**49.3**
	5.0	**72.0**	1-(5′-Bromo-4′-hydroxy-3′-methoxyphenyl)- (**28**)	1.0	**29.8**
	10.0	**82.6**		5.0	**46.1**
**1-(3′- Chlorophenyl)-** (11)	1.0	**52.7**		10.0	**69.0**
	5.0	**53.0**	1-(3′,4′-Methylenedioxyphenyl)- (**29**)	1.0	**71.1**
	10.0	**71.6**		5.0	**77.6**
**1-(4′- Chlorophenyl)-** (12)	1.0	**40.1**		10.0	**68.9**
	5.0	**67.4**		1.0	**46.8**
	10.0	**67.5**		5.0	**57.6**

**1-(2′-Bromophenyl)-** (13)	1.0	**69.5**	1-(2′-Bromo-4′,5′-Methylenedioxyphenyl)- (**30**)	10.0	**61.1**
	5.0	**70.7**	1-(4′,5′-Methylenedioxy-2′-chlorophenyl)- (**31**)	1.0	**40.6**
	10.0	**56.2**		5.0	**63.9**
**1-(3′- Bromophenyl)-** (14)	1.0	**44.6**		10.0	**73.0**
	5.0	**72.5**	1-(2′-Aminophenyl)- (**32**)	1.0	**63.9**
	10.0	**65.6**		5.0	**82.6**
1-(4′- Bromophenyl)- (15)	1.0	**77.1**		10.0	**71.0**
	5.0	**78.6**	**1-(3′-Aminophenyl)- (33)**	1.0	**81.2**
	10.0	**79.0**		5.0	**73.4**
1-(4′-Dimethylaminophenyl)- (16)	1.0	**77.6**		10.0	**55.7**
	5.0	**80.2**	1-(4′- Aminophenyl)- (**34**)	1.0	**66.0**
	10.0	**77.1**		5.0	**81.2**
				10.0	**75.6**

Table S2. Effects of the examined compounds at three doses on plasma PGE_2 levels in a carrageenan-induced inflammation model (M±SEM, n=6).

**Examined compound**	**Dose, mg/kg**	**PGE 2 level, pg/mL**	**Examined compound**	**Dose, mg/kg**	**PGE 2 level, pg/mL**
Intact		352.6 ± 15.0	**17**	1.0	654.9 ± 20.0*
				5.0	636.7 ± 23.5*
				10.0	611.2 ± 22.5*
Control carrageenan 1.0 % 0.1 ml	0.2 ml	967.2 ± 25.0	**18**	1.0	524.5 ± 19.5**
				5.0	497.1 ± 18.5**
				10.0	514.8 ± 19.0**
Dicloberl retard + carrageenan 1.0 % 100 µl	8.0	536.5 ± 20.0*	**19**	1.0	475.5 ± 17.0**
				5.0	516.7 ± 19.0**
	10.0	511.3 ± 19.0*		10.0	554.3 ± 20.5**
**2**	1.0	**495.4 ± 18.0****	**20**	1.0	502.2 ± 18.5**
	5.0	483.6 ± 17.5**		5.0	462.4 ± 17.0**
	10.0	544.2 ± 20.0**		10.0	485.9 ± 18.0**
**3**	1.0	561.5 ± 21.0**	**21**	1.0	562.5 ± 21.0**
	5.0	547.3 ± 20.0**		5.0	518.8 ± 19.0**
	10.0	542.1 ± 19.5**		10.0	493.2 ± 18.0**
**4**	1.0	588.7 ± 22.0**	**22**	1.0	638.4 ± 23.5*

	5.0	573.5 ± 21.0**		5.0	617.0 ± 22.5*
	10.0	579.9 ± 21.5**		10.0	603.6 ± 20.0*
	1.0	624.2 ± 23.0*		1.0	459.4 ± 17.0**
**5**	5.0	602.7 ± 22.0*	**23**	5.0	482.2 ± 18.0**
	10.0	591.0 ± 21.5*		10.0	545.6 ± 20.0**
	1.0	546.3 ± 20.0**		1.0	537.0 ± 20.0**
**6**	5.0	492.8 ± 18.0**	**24**	5.0	478.7 ± 17.5**
	10.0	476.1 ± 17.0**		10.0	489.5 ± 18.0**
	1.0	518.6 ± 19.0**		1.0	582.2 ± 21.5**
**7**	5.0	475.2 ± 17.0**	**25**	5.0	536.9 ± 20.0**
	10.0	461.8 ± 16.5**		10.0	501.4 ± 18.5**
	1.0	503.1 ± 18.5**		1.0	532.7 ± 19.5**
**8**	5.0	473.6 ± 17.0**	**26**	5.0	491.2 ± 18.0**
	10.0	468.4 ± 16.5**		10.0	469.5 ± 17.0**
	1.0	639.9 ± 24.0*		1.0	672.1 ± 24.5*
**9**	5.0	674.1 ± 25.0*	**27**	5.0	651.8 ± 24.0*
	10.0	698.8 ± 26.0*		10.0	638.3 ± 23.5*
	1.0	**487.1 ± 18.0****		1.0	644.4 ± 24.0*
**10**	5.0	466.3 ± 17.0**	**28**	5.0	619.2 ± 23.0*
	10.0	459.5 ± 16.5**		10.0	504.9 ± 18.5**
	1.0	582.7 ± 21.5**		1.0	498.6 ± 18.5**
**11**	5.0	527.9 ± 19.5**	**29**	**5.0**	479.1 ± 17.5**
	10.0	484.5 ± 17.5**		10.0	511.7 ± 19.0**
	1.0	705.1 ± 26.0*		1.0	649.3 ± 24.0*
**12**	5.0	694.0 ± 25.5*	**30**	5.0	631.8 ± 23.5*
	10.0	678.6 ± 25.0*		10.0	603.1 ± 22.0*
	1.0	502.9 ±18.5**		1.0	525.9 ± 19.5**
**13**	5.0	489.1 ± 18.0**	**31**	5.0	497.2 ± 18.5**
	10.0	499.7 ± 18.5**		10.0	471.5 ± 17.0**

	1.0	547.3 ± 20.0**		1.0	502.3 ± 18.5**
**14**	5.0	491.5 ± 18.0**	**32**	5.0	452.1 ± 16.5**
	10.0	508.2 ± 18.5**		10.0	475.6 ± 17.5**
	1.0	**481.4 ±17.5****		1.0	**451.4 ± 16.5****
**15**	5.0	476.0 ± 17.0**	**33**	5.0	473.7 ± 17.5**
	10.0	465.7 ± 16.5**		10.0	487.3 ± 18.0**
	1.0	512.5 ± 19.0**		1.0	479.9 ± 17.5**
**16**	5.0	485.8 ± 18.0**	**34**	5.0	456.2 ± 16.5**
	10.0	497.2 ± 18.5**		10.0	461.6 ± 17.0**

Table S3. Effects of the examined compounds at three doses on plasma TXB_2 levels in a carrageenan-induced inflammation model (M±SEM, n=6).

**Examined compound**	**Dose, mg/kg**	**Decreace in TXB_2 level %**	**Examined compound**	**Dose, mg/kg**	**Decrease in TXB_2 level, %**
		100		1.0	45.3**
Intact		174	**18**	5.0	37.7**
		52.1*		10.0	41.0**
Control carrageenan 1.0 % 0.1 ml	0.2 ml	45.0**	**19**	1.0	85.7
		40.4**		5.0	80.5
		37.1**		10.0	73.3
Dicloberl retard + carrageenan 1.0 % 100 µl	8.0	54.3*		1.0	48.7*
		59.2*	**20**	5.0	40.9**
	10.0	55.2*		10.0	46.0*
	1.0	53.7*		1.0	34.8**
**2**	5.0	66.9	**21**	5.0	46.5*
	10.0	62.6		10.0	57.2*
**3**	1.0	64.4	**22**	1.0	42.4**

	5.0	77.0		5.0	31.1**
	10.0	70.9		10.0	37.8**
	1.0	67.6		1.0	59.5*
**4**	5.0	54.9*	**23**	5.0	47.1*
	10.0	39.7**		10.0	39.8**
	1.0	35.0**		1.0	81.0
**5**	5.0	47.0*	**24**	5.0	74.9
	10.0	34.7**		10.0	71.1
	1.0	30.9**		1.0	30.2**
**6**	5.0	42.6**	**25**	5.0	36.7**
	10.0	34.3**		10.0	54.7*
	1.0	32.8**		1.0	52.2*
**7**	5.0	81.4	**26**	5.0	35.7**
	10.0	91.1		10.0	38.8**
	1.0	98.1		1.0	65.1
**8**	5.0	38.1**	**27**	5.0	52.2*
	10.0	32.2**		10.0	42.2**
	1.0	30.3**		1.0	51.0*
**9**	5.0	65.2	**28**	5.0	39.3**
	10.0	49.7*		10.0	33.1**
	1.0	37.4**		1.0	90.6
**10**	5.0	99.9	**29**	**5.0**	84.8
	10.0	96.8		10.0	81.0
	1.0	92.4		1.0	82.7
**11**	5.0	42.6**	**30**	5.0	75.6
	10.0	38.7**		10.0	43.1**
**12**	1.0	38.1**	**31**	1.0	41.4**

	5.0	32.2**		5.0	35.8**
	10.0	30.3**		10.0	45.1**
**13**	1.0	65.2	**32**	1.0	84.1
	5.0	49.7*		5.0	79.1
	10.0	37.4**		10.0	71.0
**14**	1.0	99.9	**33**	1.0	49.1*
	5.0	96.8		5.0	41.0**
	10.0	92.4		10.0	33.7**
**15**	1.0	42.6**	**34**	1.0	42.4**
	5.0	38.7**		5.0	28.2**
	10.0	41.5**		10.0	34.8**
**16**	1.0	55.2*	35	1.0	28.0**
	5.0	39.3**		5.0	34.3**
	10.0	44.1**		10.0	38.2**
**17**	1.0	36.5**	36	1.0	36.1**
	5.0	34.9**		5.0	29.3**
	10.0	32.0**		10.0	30.9**
